# Heterogeneities in landed costs of traded grains and oilseeds contribute to unequal access to food

**DOI:** 10.1038/s43016-024-01087-7

**Published:** 2025-01-06

**Authors:** Jasper Verschuur, Yiorgos Vittis, Michael Obersteiner, Jim W. Hall

**Affiliations:** 1 Oxford Programme for Sustainable Infrastructure Systems, Environmental Change Institute, University of Oxford, Oxford, UK; 2 Faculty of Technology, Policy and Management, Delft University of Technology, Delft, the Netherlands; 3 Ecosystem Services and Management Program, International Institute for Applied Systems Analysis, Laxenburg, Austria; 4 Environmental Change Institute, University of Oxford, Oxford, UK

**Keywords:** Economics, Environmental impact, Geography, Agriculture

## Abstract

Despite the growing accessibility of international grain and oilseed markets, high production costs and trade frictions are still prevalent, contributing to regional heterogeneities in the landed cost of grain imports. Here we quantify the landed cost for six grain commodities across 3,500 administrative regions, capturing regional cost differences to produce grain and transport it across international borders. We find large heterogeneities in the costs of imported grain, which are highest in Oceania, Central America and landlocked Africa. While some regions have uniform landed costs across sourcing locations, others face cost variations across trading partners, showing large inequalities in access. We find that most regions could benefit from a targeted approach to reduce landed cost while others benefit from a mixed strategies approach. Our results highlight that spatial information on production, trade and transport is essential to inform policies aiming to build an efficient and resilient global agricultural commodity trade system.

## Abstract (web summary)

The cost of transporting agricultural products from the field to consumers across borders, also known as the landed cost, is highly influenced by trade frictions and regional cost differences. This study estimates this cost—including production, transport and trade components—for six grain commodities across 3,500 administrative regions, revealing large inequalities in access.

## Main

Between 1995 and 2018, global agricultural and food trade has more than doubled^1^, with 20% of globally consumed calories derived from food imports^2^. This has contributed to reduced food insecurity globally by connecting surplus regions with those of deficit, ensuring year-round access to food, mitigating potential weather-related local food input price shocks and lowering food prices^3–5^.

Food security comprises four key pillars: food availability, food access, food utilization and their stability^6^. Research on food security has extensively focused on the link between international trade and its contribution to food availability, including the role of trade to cope with climate change impacts on global security^7–9^. By contrast, there has been considerably less emphasis on understanding the impact of changes in cost drivers of internationally traded food^10^ and the differences across importers, which determine access and affordability. Yet, it is widely known that the costs to source imported food differ considerably across countries^8,10^, and even within countries^11,12^.

Cost heterogeneities of food imports are driven by several factors, including differences in the production costs across exporting regions, tariffs and trade agreements, transport costs, border compliance costs and non-tariff barriers^8,10,13–15^. Previous research has shown that production costs for specific agricultural commodities can differ by a factor five to ten across countries, given variations in chemical input (pesticides and fertilizer), labour and machinery costs and commodity yields^14^. In addition, the cost to transport goods from field to customer are known to be important in agricultural trade, given the relatively low value-to-weight ratio of agricultural products and long distances between trading partners. For instance, maritime transport costs are found to be on average 5–15% of the total cost at the importing port (that is, the cost from field to importing port) of grains and oilseeds, but can differ across trading pairs^13^. On top of that, hinterland transportation costs can add to the total transport costs, in particular in developing and landlocked countries^16–18^. High transport costs can be an important trade friction that can limit trade^10^, preventing certain regions from reaping the benefits of access to international markets.

So far, there is limited understanding of the magnitude and spatial distribution of the landed costs of international agricultural trade, defined as the cost to source agricultural products from field to consumers across borders. Moreover, little is known about the respective cost breakdown of the landed cost into production costs components, transport costs and trade costs (that is, border compliance costs and tariffs). For instance, previous studies have evaluated the transport distances between global grain production and consumption regions based on transport friction surfaces^19^. However, the use of transport friction surfaces ignores the availability of transport connections (for example, whether ports are connected by shipping lines), the quality transportation infrastructure and the cost associated with transport distances and mode changes, which are all key determinant for market accessibility. Others have tried to quantify the importance of maritime transport costs (in total sourcing costs) for grains and oilseeds using the difference between ‘free on board’ and ‘cost, insurance and freight’ prices^13^. However, the hinterland transport costs cannot be derived from this, nor the production costs, as well as data availability being relatively limited. Others, such as Janssen et al.^10^, have tried to construct a detailed representation of aggregate trade costs between subnational regions, in this case within the African continent and trading partners outside the continent, which was part of a comprehensive modelling study to evaluate shifting food trade patterns across the African continent as a result of lowering trade barriers and transport costs. However, so far, none of the presented methodologies allow for a detailed breakdown of various cost drivers on a global scale, nor have intended to capture a detailed physical representation of the global (multimodal) transport network.

As such, there is considerable scope to complement existing studies and perform highly granular analyses of the total landed cost and cost breakdown of internationally traded agricultural products using a detailed global transport model. This information is essential for studying the global impacts of national and international food policy reforms. First, it would help to quantify geographical disparities to access international food markets. Second, it could support the identification of strategies to improve market access and reduce the cost of imports, through productivity enhancements in the exporting country, investment in transport infrastructure, trade facilitation and tariff policy reform. Third, it would allow predicting how input price shocks to any or several of these cost components can propagate through international trade networks and affect consumers in importing countries. This is particularly relevant at the backdrop of record-high food price spikes in 2022–2023 given price increases of production input (for example, fertilizer and pesticides)^20^. A granular spatial analysis of production and trade costs would be particularly relevant for grain commodities, which are particularly affected by cost fluctuations, are widely traded and, for transport costs, are particularly relevant given the low value-to-weight ratio of these agricultural commodities.

In this study, we quantify the magnitude of the landed cost of six grain and oilseed (hereafter simply grains) commodities across 3,500 subnational administrative regions and provide a detailed breakdown into major cost drivers separated by transport, trade (for example, tariffs and border compliance) and production costs. We do this based on the global trade patterns reflecting the current international sourcing patterns (2017–2021 average) for maize, wheat, sorghum, barley, soybean and rice, selected because of their importance for global calorie intake and livestock feed supply (Supplementary Table 1). We further utilize the Global Landed Cost Model (G-LCM), developed as part of this study (Methods and Supplementary Fig. 1), to (1) evaluate the transmission of a hypothetical input price shock, which mimics the shock experienced in 2022 as a result of rising prices of production inputs, such as diesel, fertilizer, pesticides and transport costs, through the international grain trade network, and (2) identify hotspot regions for landed cost reductions and provide insights into the factors driving potential cost savings.

## Results

The G-LCM model estimate the landed costs for ~9 million trade dependencies between administrative regions globally, based on ~20,000 bilateral trade flows. These landed costs of imported grains can be subdivided in five production cost components (that is, fertilizer, pesticides, machinery, labour and diesel for farm machinery), storage costs, transport costs (costs and time for (un)loading, road and rail transport, port handling and maritime transport), border and custom compliance costs and import tariffs, providing a breakdown of nine cost components. It should be noted that all results hereafter refer to costs, not prices, as price mark-ups and subsidies are not included, nor how the costs to source from different regions translate into (equilibrium) consumer prices.

### Global grain commodity trade

We find an average yearly trade of 660.2 million tonnes of the six grain commodities in our trade dataset. The total freight generated to transport these six food commodities equals around 7.45 trillion tonnes per kilometre (that is, distance multiplied by quantity), with a weighted average field-to-customer distance of 11,281 km. The vast majority of freight is associated with soybean trade (around 3.0 trillion tonnes per km), followed by maize and wheat (1.9 and 1.7 trillion tonnes per km, respectively). The field-to-customer transport distance of soybean is, on average, twice that of wheat (~8,000 km) and maize (~10,000 km) owing to long-distance imports by East Asian countries. An estimated 86.0% of this trade takes place via maritime transport as the main mode, while rail (9.3%) and road (4.7%) are responsible for the remainder. In tonnes per kilometre terms, however, the share of maritime transport reaches 98.4%, emphasizing its dominance in long-distance transport.

### Global landed costs of international trade

**Fig. 1:**
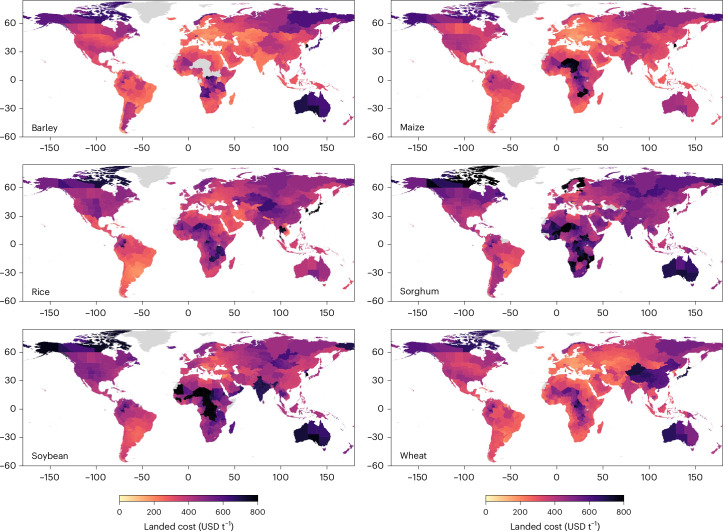
Global landed cost per commodity type. The weighted average (weighted by import quantity) landed cost per commodity type for the 2017–2021 trade network. The grey regions do not trade this specific commodity or are not included in the dataset. The basemap is from GADM (gadm.org).

Figure 1 shows the total landed cost across administrative regions globally per grain commodity. The global import weighted landed cost is lowest for barley (288.7 USD t^−1^), wheat (302.0 USD t^−1^) and maize (322.6 USD t^−1^), and highest for soybeans (385.8 USD t^−1^), rice (404.4 USD t^−1^) and sorghum (442.0 USD t^−1^). However, strong regional differences are present (Supplementary Table 2): 5.0–11.2% of administrative regions have landed costs worth 1.5 times the global average and, for certain commodities (soybeans, sorghum and rice), 1.0% or more of administrative regions have landed costs worth three times the global average.

The highest landed costs are faced by administrative regions in Oceania, Central America, sub-Saharan Africa and East Asia. However, different cost drivers account for high landed cost. In East Asia, high import tariffs (for example, in South Korea and Japan) and long-distance food imports of grain commodities contribute to high landed costs (for example, soybean imports from South America to China). In Central America and sub-Saharan Africa, the high cost of road and rail add significantly to the landed cost, particularly in landlocked countries. In Oceania, the high shipping costs and geographical distance from grain markets add to the landed cost. On the other hand, Brazil, Northern Africa, Eastern Europe and Central Asia have considerably lower landed costs given their proximity to major grain production regions and large imports of grain commodities with lower landed costs (for example, wheat, maize and barley). Supplementary Fig. 2 shows the weighted average transport cost across the trading partners and commodity types, while Supplementary Figs. 3–5 show the average field-to-customer distance (Supplementary Fig. 3), the share of landed costs associated with transport (Supplementary Fig. 4) and the share of landed costs associated with production (Supplementary Fig. 5).

### Inequality in landed costs across suppliers

**Fig. 2:**
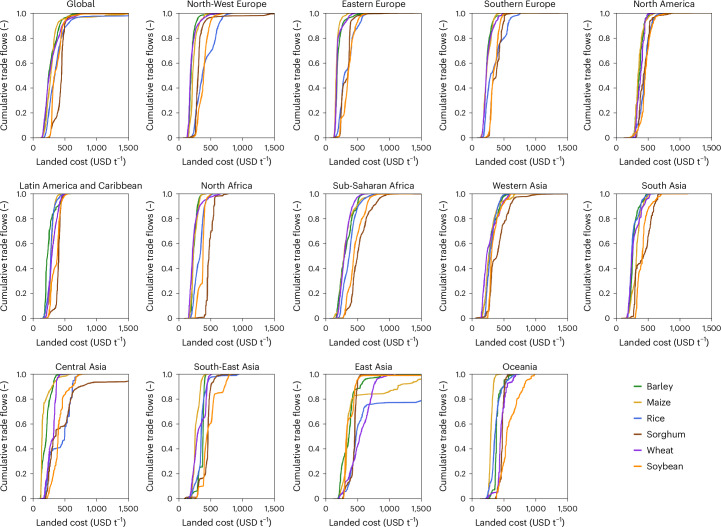
Cumulative distribution plots of landed costs across geographies. Graphs showing the distribution of the landed costs across all the cumulative trade flows modelled across administrative regions within the respective geographical areas, sorted by the landed costs. Steeper vertical import supply cost curves indicate lower inequality, whereas more horizontal curves indicate large inequality.

The weighted average landed costs hide the fact that part of the international grain trade can be sourced at much lower costs, while another part needs to be sourced at much higher costs. We can illustrate this using the cumulative distribution functions of the landed cost per geography and grain commodity (Fig. 2). On the basis of the supply cost curves, we can quantify the inequality of sourcing costs (ISC), which we define as the difference between the 90th percentile (Q90) and the 10th percentile (Q10) divided by the 50th percentile (Q50) costs for a particular importing geography and grain commodity. Higher inequality indicates large relative differences between the costs associated with the cheapest 10% sourced versus the costs associated with the most expensive 10% sourced. Globally, the ISC is low for sorghum and soybean and (0.43 and 0.53, respectively), while high for barley and rice (0.96 and 1.11, respectively).

Some geographies (see Supplementary Table 3 for the ISC per geography) generally have relatively steep and narrow supply cost curves (Southern Europe, North America, Latin America, North Africa and West and South Asia) for most grain commodities, reflecting that the differences between the cheapest and most expensive sourcing locations are relatively small. Other geographies have wider supply cost curves (sub-Saharan Africa, Central Asia, East Asia and Oceania). In other words, while some of the imports can be sourced at low cost (for example, trading partners are close by), the remaining imports to close the import demand is met by sourcing from trading partners at higher costs. For all these geographies with high ISC, the ISC is close to one or larger, indicating that the difference in landed costs between Q90 and Q10 is equal (or more) than the landed cost of Q50. As such, improving market access would involve both lowering the average landed costs and reducing the inequality in landed costs.

### Cost breakdown of landed costs

**Fig. 3:**
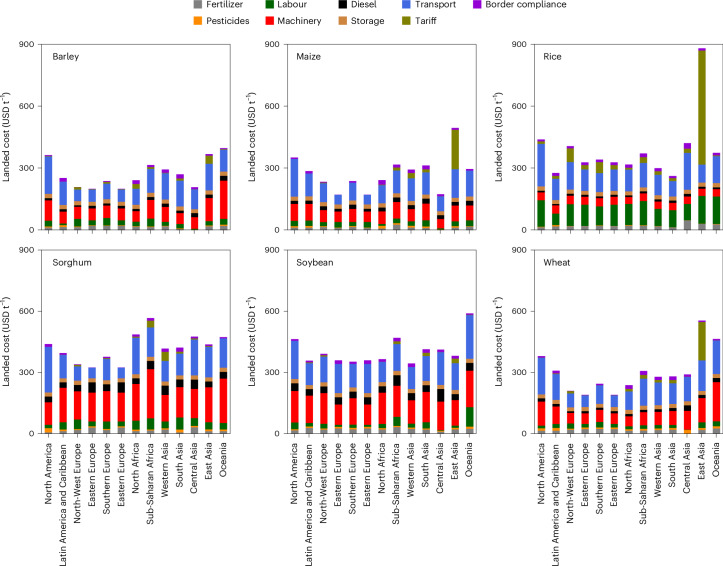
Regional cost breakdown of landed cost. The cost breakdown per import region, showing the contribution of nine cost components considered in this study to the weighted average landed cost. Diesel here reflects the diesel used for farm machinery.

The landed cost can be split into nine cost drivers, which vary across the grain commodities and different geographies (Fig. 3). The differences in landed cost between commodities are mainly driven by differences in production costs, while differences across geographies are driven by variations in transport costs and tariffs. Sorghum and soybean production involve high machinery costs and diesel input, which are propagated into the landed costs, while rice production is labour intensive. Although the transport and border compliance costs are relatively equal across grain commodities in absolute terms, their relative contributions differ across geographies and commodities. Globally, the share of transport cost to landed cost is around 25.8–36.6%, while the share of production to landed cost is 47.7–64.4% (the remaining being storage, border compliance and tariffs).

Regionally, the breakdown of the different cost drivers can vary strongly. Across grain commodities, production cost is the dominant contribution to landed cost for 64.5–90.1% of administrative regions. However, transport costs can contribute to over 50% of landed cost in some administrative regions (Supplementary Fig. 4), and is even the dominant cost driver for 6–11% of administrative regions (for barley, maize and wheat), most prominently in Latin America, sub-Saharan Africa and Western Asia.

A further breakdown of the transport cost in the maritime transport cost and land transport cost (road and rail), as a weighted average across all grain commodities (Supplementary Fig. 6), shows that differences in maritime transport costs are more country specific, driven by the trade network of the country (which determines the average distance travelled) and the unit maritime transport cost (in USD per tonne per kilometre). The latter is determined by a country’s maritime connectivity (for example, direct or indirect connections) and the vessel fleet calling at ports (for example, whether grains can be transported in bulk and the size of maritime vessels calling), and can vary by a factor of two between the median and 90th percentile subnational administrative region. The land transport cost, which is the dominant source of total transport costs for 98% of subnational regions, varies more strongly across subnational regions, explaining most of the variation in landed cost within countries. Despite much lower distances travelled (median distance being 1,536 km for land versus 5,616 km for maritime), the median unit costs of land-based transport is 16.5 times higher compared with the unit maritime transport cost, and varies equally strong across subnational regions (the 90th percentile being 1.4 times the median value). In particular, in the aforementioned places where transport costs are dominant (landlocked subnational regions of Latin America, sub-Saharan Africa and Western Asia), a combination of long land-based transport distances (Latin America and Western Asia) and high unit transport costs (sub-Saharan Africa) result in high land-based transport costs.

In other cases, the contribution of transport cost is only a small fraction of the landed costs, for instance in East Asia (rice and wheat), Eastern Europe (all commodities except sorghum), South America (soybean, maize and sorghum) and South Asia (sorghum). In these places, the low contribution of transport is driven by the close proximity to grain markets (Supplementary Fig. 3), although the small contribution of transport cost in East Asia is related to the high import tariff set on commodities, which dominates the cost breakdown.

### Transmission of an input price shock

The relative importance of different cost components in the landed costs is essential information to understand how global, or geographically specific, input price shocks to any of the cost components can be transmitted through the grain trade network in case the cost increase is passed on to the consumers (that is, cost pass-through). We evaluate how administrative regions are exposed to an input price shock resembling conditions in 2022 (mostly due to the Russian invasion of Ukraine) for diesel (+80%), transport (+50%) and chemical input (+200% for fertilizer and pesticides) (Supplementary Table 6). The diesel price increase reflects the diesel input for farm machinery, whereas the transport cost increase indirectly reflects the increase in the global oil price, but also other factors (for example, demand and supply-side effects).

Here, we show how the aforementioned cost heterogeneities determine the cost pass-through, and how different subnational regions are exposed to it. It should be noted that this is a static representation without accounting for dynamic adjustments in the production system (for example, changing fertilizer input or crop switching), trade patterns (for example, switching suppliers) or demand (for example, reduced demand), and it is not intended to model the impact on global or national food prices.

**Fig. 4:**
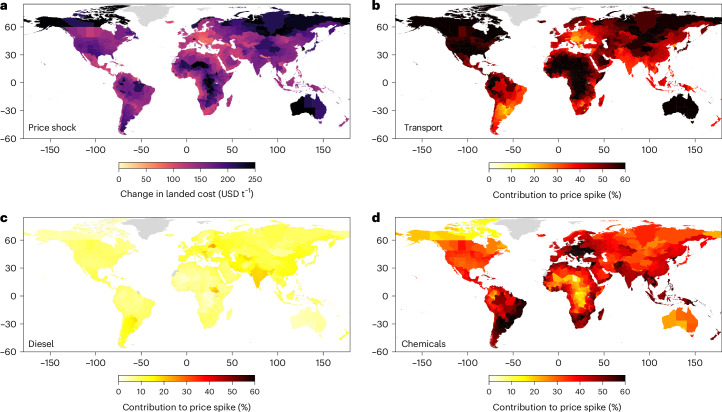
Increase in landed costs due to input price shocks by cost component. **a**, The total increase in landed costs (USD t^−1^) due to the combined input price shock implemented. **b**–**d**, The share of transport (**b**), diesel (**c**) and chemical (pesticides and fertilizers) (**d**) costs contributing to the total input price shock cover. The basemap is from GADM (gadm.org). Diesel here reflects the diesel used for farm machinery.

Globally, we estimate this weighted average cost increment in landed cost to be 42.8% (+144.0 USD t^−1^) (Fig. 4a and Supplementary Fig. 7 showing the relative change). Some of the largest relative changes are observed in South America, the Baltic states, Central Asia and countries in Southern Africa (Lesotho, Namibia, Botswana and Eswatini), with cost increases of over 50%. This is mainly related to low landed costs to start with (given proximity to grain markets), which makes the cost pass-through (in relative terms) high.

Figure 4b–d shows the contribution of the different cost components to total landed costs. The contribution of the increase in diesel price is limited (between 10% and 20%), with the remaining being a split between the transport and chemical cost increase. The chemical input cost increase is the dominant driver for 58% of administrative regions, while for the remaining 41%, the increase in transport cost is the dominant driver. This underlines that subnational regions might be exposed differently to specific input cost fluctuations, requiring granular data on the cost drivers of each administrative region.

**Fig. 5:**
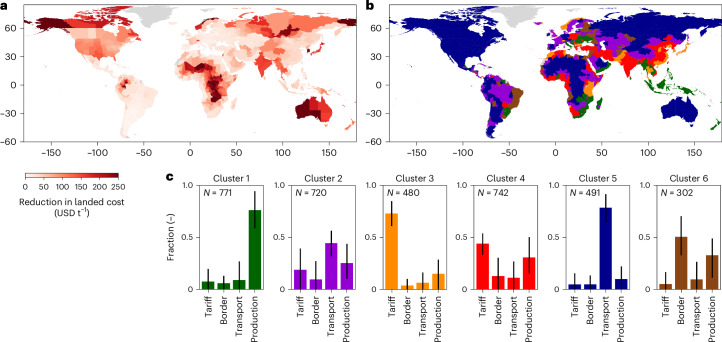
Strategies to reduce the cost of imported grains and oilseeds. **a**, The absolute reduction in the weighted average landed cost across the six commodities. **b**, Clustering of regions in terms of the potential strategies to reduce the cost of imported food. **c**, An overview of the contribution of the four cost components to the overall cost reduction per cluster. The basemap is from GADM (gadm.org).

### Strategies to reduce the landed cost of grain imports

The stark regional heterogeneity of landed costs indicates that there is potential to reduce these costs, for instance through reducing tariffs (for example, tariff reforms), smoothing border and customs processes (for example, digitalization), lowering transport costs (for example, investment to improve the efficiency of transportation) and improving agricultural productivity (for example, close yield gaps, better land-use practises and investments in automation and machinery).

We use our global landed cost analysis to identify places where there is potential for cost reductions by lowering some of the costs to those representing the median across all administrative regions (Supplementary Table 7). We then propagate these cost reductions through the international trade network and see how it would affect the weighted average landed costs across the commodities considered. Moreover, we use a clustering algorithm (*k*-means; Methods) to identify six cost reduction archetypes, identifying the most promising strategy to adopt for every administrative region. While our method does not consider the feasibility of achieving these reductions, the results can serve as a starting point for policymakers to identify existing cost barriers to improve access to international markets.

Figure 5a shows the reduction in landed costs that could be achieved per administrative region. Globally, the weighted average reduction in landed costs equals 57.3 USD t^−1^ (17% reduction), although some regions have much higher potential. These high potential administrative regions (landlocked regions in Africa and central America) have multiple cost contributions that are higher compared with the global average, and hence have potential to be reduced over time. Cluster 1 captures administrative regions that source from exporting countries with above-average production costs (Eastern Europe and South-East Asia; Fig. 5b). Agricultural improvements in these production countries, where possible given technical, social and natural constraints, would yield benefits in terms of reducing the cost of grain imports. Cluster 3 includes countries in South and East Asia, Norway and East Africa, which impose high import tariffs on their imported grains. While raising revenue and protecting domestic markets, they also place a burden on the affordability of imported grains. Clusters 2 and 4 encompass regions where cost reductions can be achieved via a mix of measures, and hence focusing on one of the four might not yield the desired benefit. Cluster 5 is those regions where the above-average transport cost is the main barrier, which are located in parts of landlocked Africa, Central Asia, North America, Australia and South America. This is driven by the relatively long port-to-customer distances, which can drive up transport costs, and hence can benefit from investments in transport. Cluster 6 includes regions that generally have low potential for absolute cost reductions (Fig. 5a), but where border compliance costs could be reduced via trade facilitation strategies.

## Discussion

Despite the growing integration of international grain markets, the costs to source grain commodities still varies dramatically around the world. In this study, using a newly developed G-LCM, we have sought to explain some of these differences in the costs to sourcing grains (called the landed cost) by quantifying the main contributors to the cost of grain production, transport and trade.

Countries in landlocked sub-Saharan Africa, Central America, East Asia and Oceania face disproportionately high landed costs, though the factors that drive these costs differ. For instance, countries in the Pacific face large landed costs given the distance to export markets and resulting high cost of maritime transport. Landlocked countries in sub-Saharan Africa and Central America have disproportionately high hinterland transport costs that add to the total landed cost. In East Asia, high import tariffs add up significantly to landed costs. We have identified region-specific cost reduction strategies that could lower grain import costs, though for some regions, several policies will need to be applied if grain imports are to be made more affordable. Further research is needed that captures how such cost-reduction strategies can be designed and what natural, technical, social or political-economical barriers exist to achieving them. Historical data capturing ways in which countries have tried, or are trying, to lower their landed costs, and the benefits and costs of these investments or policy reforms, can help in achieving this.

We further show how there are high inequalities in the cost of grain imports, expressed as the differences between the landed costs to source the first ten per cent versus the last ten per cent of imports. In other words, focusing solely on the weighted mean costs hides the differences in importing grains from the variety of exporting countries that a country sources from. High inequalities can be caused by sourcing from a set of trading partners, part of which are constrained in their export quantity, a lack of access to large export markets (with lower costs) or large differences in trade barriers between exporting countries such as tariffs. Therefore, alongside lowering overall landed costs, reducing inequalities in landed costs should be seen as a priority to enhance access to international markets and achieve a more equitable trade network. However, the benefits of accessing new markets should be weighed against the exposure to shocks in those new exporting regions, making sure that improving access does not come at the expense of increased vulnerability.

By adding a hypothetical input price shock to the model, reflecting the conditions experienced in 2022, we highlight how different countries are exposed to such a shock (that is, cost pass-through). While globally we find a landed cost increase of 144 USD t^−1^ (+42.8%), above-average increases in landed costs are found in landlocked countries in Africa, the USA, Australia, Central Asia and Russia. The relative importance of chemical input in the total production costs, as well as the distance that goods are shipped, is a predictor for differences in the increase in landed costs. As such, this framework can provide the basis for evaluating a country’s exposure to (short-term) food input price shocks, and how the increasing costs of imports can contribute to domestic food price inflation (that is, the pass-through effect of higher import costs)^21^. This can feed into strategies to cope with price spikes, for example, through government or internationally financed support schemes. Moreover, it can form the basis for modelling studies that aim to quantify the dynamic response of the global supply system to such an input price spike (for example, using a partial equilibrium model), which include supply, trade and demand adjustments as a result of a cost increase or decrease (for example, investments).

As with all global analysis, some modelling shortcomings should be highlighted. Most importantly, our modelling framework does not account for price mark-ups throughout the supply chain or government subsides of imported staple foods, as well as price fluctuations due to demand and supply variations. Moreover, some fixed-cost elements are not included in the production costs estimates, such as land values, insurance and financing costs, given the lack of data, despite being important cost factors in certain countries. Instead, we provide a static representation of the costs associated with the field-to-customer chain, with consumer demand scaled by population size, on top of which additional cost components could be added.

The future outlook of global grain commodity supply is uncertain as a result of geopolitical fragmentation and the impacts of climate change on global yields. However, we have shown that several of the drivers of landed costs are a consequence of inefficiencies that could be ameliorated through improving trade facilitation, tariff reforms and reducing transport costs. Furthermore, while occasional shocks to the cost of input factors may be inevitable, our analysis helps to identify places that will be disproportionately impacted by a given shock, which can be used to target mitigation interventions.

## Methods

The methodology can be separated in three main model components of the G-LCM: (1) the global production cost component, (2) the global transport cost component and (3) the (subnational) trade flow allocation component. After this, we describe two types of scenarios used in the analysis: (1) the implementation of the input price shock and (2) the implementation of the cost-reduction strategies. An overview of the workflow is shown in Supplementary Fig. 1.

### Global production cost component

The global production cost and trade components represent where certain grain commodities are produced, at what costs and which countries consume these grains. We are looking at administrative regions globally (the first administrative unit, that is, province or state), as this is a compromise between adding detail in regional differences in production and costs, while keeping it aggregated to allow the model to be computationally tractable. Subnational regions are based on the Global Administrative Areas (GADM) database (https://gadm.org/), together resulting in ~3,500 regions of interest.

For the six grain commodities under consideration, we add, per administrative region (*r*), the commodity production and commodity yields, both derived from spatially explicit commodity maps^22^. To estimate regional differences in grain imports, we add population counts (Pop) per administrative region, derived from the Global Population of the World (GPWv4) dataset^23^, assuming that the demand for commodity imports scales with the number of people living in administrative regions (relative to the total country population). The commodity demand captures both human consumption of food, food required to feed livestock and other uses of grains (for example, processing, losses, seed and other food uses). We can thus write the consumption of grain import per grain commodity (*g*) as

$${C}_{r,g}={I}_{c,g}\times \frac{\mathrm{{Po}{p}}_{r,g}}{\sum _{r}{\mathrm{{Po}{p}}}_{r,g}},$$

with *I* being the total imports of a specific grain commodity for the country (*c*) the administrative regions are part of. We extract country-to-country trade data for the years 2017–2021 from the 'Base Pour L'Analyse Du Commerce International' (BACI) trade database, which is a harmonized trade dataset based on UN Comtrade data^24^. Given the year-to-year variability in grains trade flows, we average trade flows over this five-year period to create a representative trade network for the six grain commodities, which smooths out fluctuations between trade across years. For countries where grain imports will mainly be used to feed livestock, the assumption of scaling import demand with population counts may be biased. Though a necessary assumption, Supplementary Fig. 9 shows the share of feed consumption across all human and non-human commodity consumption.

Per commodity type and country, we add production costs for five input components (fertilizer, pesticides, labour, machinery and diesel) expressed in USD per hectare (in constant 2020 values). These data are derived from previous work, which developed a bottom-up agricultural cost engineering model of commodity-specific farming input for 146 commodity-producing countries^14^. Although covering the largest share of the production costs, some fixed costs are omitted, such as costs associated with land, insurance, buildings and financing, which are context specific and can vary widely across countries and across farms within countries.

By dividing the per hectare cost with the commodity yield (*Y*) per administrative region, we estimate the regionally varying production cost (PC) per administrative region as

$${\mathrm{PC}}_{r,g}\left({\mathrm{USD}}/{\mathrm{t}}\right)={\mathrm{CF}}\times \frac{\mathrm{PC}_{c,g}\left({\mathrm{USD}}/{\mathrm{ha}}\right)}{{Y}_{r,g}},$$

with CF being a correction factor, which reflects that, in practise, this relationship is not linear, as high-yielding regions might have higher input costs compared with low-yielding regions. On the basis of detailed farm surveys in the USA^25^, India^26^ and the EU^27^, we derive a cost-correction factor as a function of the administrative region’s yield and the country-wide median yield, as presented in Supplementary Methods 1. The resulting regional production cost estimates are included in Supplementary Fig. 8.

On top of the production costs, we also add a cost component reflecting the storage and upcountry handling cost that shippers incur before goods are exported. On the basis of indicative values from a range of countries (Canada, Australia, Ukraine, Russia, Argentina and the USA), which vary between 11 and 31 USD t^−1^, we adopt a value of 20 USD t^−1^ uniformly across countries and commodities^28^.

### Transport cost component

We created a global multimodal transport network, comprised of road, rail and maritime transport networks (Supplementary Table 4). Road and rail network data are extracted from OpenStreetMap (global planet file extracted as per November 2022), which include the speed and distance on every road segment. The speed is gap-filled per country based on the road type (for example, motorway, trunk, primary, secondary or tertiary road). The global maritime transport network comprises ~1,400 ports and the maritime connections between these ports based on 2 years of vessel movement data (2019–2020), which include the vessel type, maximum carrying capacity and actual carrying capacity (maximum carrying capacity times the payload) of vessels on every route. We split the maritime transport network into three separate transport networks based on the specific vessel types used for shipping of grain products (dry bulk, container and general cargo), as they use different terminals, have different transport costs and have varying spatial network characteristics^29,30^. Details of the network creation is provided in previous work^30^.

We add several transport components (both cost and time components) to the respective transport networks. These include distance transport costs, (un)loading times and costs, port handling costs and dwell time, vessel turnaround time, border crossing time and customs compliance costs, all of which vary between countries. While in some cases we have applied uniform values across groups of countries, in other cases we set up specific regression formulas to gap-fill country information. An overview of the different components, the source data and extrapolation (beyond country coverage) are provided in Supplementary Table 5 and explained in more detail in Supplementary Methods 2.

Using the global transport network and transport time and costs, we can estimate the least-cost transport routes between administrative regions globally. In general, freight costs comprise both distance and time-related costs^31,32^, which together inform the decision on the most likely route taken for transport^33^. Therefore, for the least-cost route optimization, we adopt a generalized cost function that includes the above-mentioned transport cost and time components, with the time components translated into equivalent costs using a value of freight travel time (VFTT). The VFTT can interpreted as the marginal rate of substitution between travel time and costs, which differ between countries, modes of transport and the actor responsible for transporting the good (for example, shipper or carrier)^31^.

The generalized cost (GC) function adopted in this study for a country (*c*) and mode (*m*) reads

$${\mathrm{GC}}_{c,m}=\underbrace{{\mathrm{DC}}_{c,m}\times{D}_{m}+{\mathrm{HC}}_{c,m}}_{{\mathrm{Transport}}\; {\mathrm{cost}}}+{\mathrm{VFTT}}_{c,m}\times\underbrace{\left({T}_{m}+{W}_{c,m}\right)}_{{\mathrm{Transport}}\;{\mathrm{time}}}+\underbrace{{B}_{c,m}}_{{\mathrm{Border}}\;{\mathrm{compliance}}\;{\mathrm{cost}}},$$

where DC is the distance costs (USD per tonne per kilometre), *D* is the travel distance (km), *T* is the travel time, *W* is the waiting or dwell time (at ports, borders, origin, destination or rail terminal), HC is the handling costs (port handling or (un)loading at origin, destination or rail terminal) and *B* is the border compliance costs. The latter includes all the cost of custom clearance (for example, documentation, inspection costs and so on).

Using this GC function, we derive the least (generalized) cost routes between administrative regions globally based on a Dijkstra shortest path algorithm^34^. This results in a GC between around 12 million pairs of administrative regions and the corresponding transport costs, transport time, distance and border compliance cost for every least-cost route. We do this separately for road transport (where possible), rail transport (where possible) and for the three maritime transport networks. The trade cost (TC) between any between exporting (er) and importing (ir) administrative region is found by adding the total transport cost from field to customer (TrC) and *B*

$${\mathrm{T{C}}}_{\mathrm{er},{\mathrm{ir}},m}={\mathrm{Tr}\mathrm{C}}_{\mathrm{{er}},{\mathrm{ir}},m}+{B}_{\mathrm{er},{\mathrm{ir}},m}.$$

For every pair of administrative regions, we select the mode with the lowest generalized cost as the representative mode to ship grains between regions, and extract the trade cost for that mode of transport.

### Trade flow allocation component

To estimate the landed costs per administrative region, we have to estimate which other administrative regions they source grains from. To do this, we scale bilateral trade flows to pairs of administrative regions to better capture from which exporting administrative regions the importing administrative regions source their commodities. We do this based on a network-based radiation model^35,36^, as opposed to the commonly applied gravity model^37^ or simple proportionate allocation method (see Supplementary Methods 3 for a discussion), since the radiation model is a parameter-free flow estimation algorithm, which therefore does not need calibration or an assumption on a distance-decay parameter. The radiation model has recently been adopted in China for freight demand modelling, showing better performance in predicting flows compared with gravity modelling^38^. Moreover, the radiation model, as adopted here, uses information on grain production, grain demand and the landed cost information that we derive, compared with simply using production and demand only (proportionate allocation) or trade distance (gravity) (Supplementary Methods 3). The rationale of the radiation model, as adopted in this paper, is that the amount exported from the exporting administrative region to the importing administrative region depends on the share of the country-wide commodity production located in the administrative regions from which it costs less to source from. In other words, importers try to minimize the landed costs of their imports from the possible exporting administrative regions.

The landed cost (LC) between any pair of administrative regions (the producing and the importing) is found by adding the commodity production cost (PC) and storage cost (ST) with the transport cost, border compliance costs and import tariffs (IT) to ship between exporting (er) and importing (ir) administrative region

$${\mathrm{L{C}}}_{\mathrm{{er},{ir}},g}={\mathrm{P{C}}}_{\mathrm{er},g}+{\mathrm{S{T}}}_{\mathrm{er},g}+{\mathrm{T{C}}}_{\mathrm{{er},{ir}}}+{\mathrm{I{T}}}_{\mathrm{{er},{ir}},g}.$$

Import tariffs are taken from the 2019 MAcMap-HS6 database from CEPII (ref. ^39^), which includes ad valorem import tariffs *(*avit) per commodity type (HS6) and trading pair (export country, ec, and import country, ic). The import tariffs are often charged on the landed cost at the port (LPC*)*, which includes the commodity production cost, storage cost, the border compliance cost in the exporting country (*B*_e_) and total transport costs from the exporting country up until the border of the importing country (TrCB)

$${\mathrm{I{T}}}_{\mathrm{{er},{ir}},g}={\mathrm{{avi}{t}}}_{\mathrm{{ec},{ic}},g}\times \left({\mathrm{P{C}}}_{\mathrm{er}}+{\mathrm{ST}}_{\mathrm{er}}+{B}_{\mathrm{{er},{ir}}}+{\mathrm{TrCB}}_{\mathrm{{er},{ir}}}\right).$$

The radiation model estimates the flow utility between regions based on the representative weights at the origin and destination administrative regions, and the friction between regions. The weight of the exporting region (*e*) is the agricultural commodity production (*P*) of the specific commodity, while the weight of the importing (*i*) administrative regions is based on the import demand (*C*). We hereby assume that the grain import demand within a country scales with population size, which may not be representative for countries that import grains primarily for feedstock. In addition, we do not distinguish between within-country access to imported grains. In many countries, imported grains will be predominantly consumed in urban regions whereas rural regions may be more reliant on domestically produced grains for consumption. These two assumptions could be refined in future work. In our specification of the radiation model, we normalize both the production and population data, as we are not interested in absolute flow prediction but merely the flow utilities. We can write the downscaled trade flows between pairs of administrative regions (*T*_ec,ic_) as

$${T}_{\mathrm{{er},{ir}},g}={T}_{\mathrm{{ec},{ic}},g}\frac{\overline{{C}_{\mathrm{ir},g}}\overline{{P}_{\mathrm{er},g}}}{\left(\overline{{C}_{\mathrm{ir},g}}+\overline{{P}_{\mathrm{{er},{ir}},g}}\right)\left(\overline{{C}_{\mathrm{ir},g}}+\overline{{P}_{\mathrm{er},g}}+\overline{{P}_{\mathrm{{er},{ir}},g}}\right)},$$

with *T*_ec,ic*,g*_ being the bilateral trade quantity and $\overline{{P}_{\mathrm{{er},{ir}},g}}$ being the commodity production in administrative regions with a LC less than the LC to ship from the exporting to the importing administrative region. This allows us to predict the flows per grain commodity and pair of administrative regions.

The weighted average landed cost per importing administrative region can then be estimated based on the quantities and landed cost across the range of exporting administrative regions an importing administrative region sources from.

### Input price shock model implementation

We calculate the impact of a representative input price shock by changing the specific cost components of the total landed cost, keeping all else equal. In other words, we evaluate what happens to the landed cost if the input price shock is fully passed on to consumers in a static fashion, not taking into account trade (for example, shifting suppliers), supply (for example, increase domestic production) or demand (demand reduction or substitution) adjustments. For the input price shock included, we look at the changes in the cost of fertilizers, pesticides, diesel for farm machinery and transport, with the values adopted as presented in Supplementary Table 6. We re-evaluated the weighted average landed cost per importing administrative region to quantify how such an input price shock is propagated through the trade network and affect administrative regions differently.

### Cost reduction strategies implementation

Per importing administrative region, we evaluate whether there is potential to reduce the cost of imported food. We look at four cost components only, which are directly linked to specific policies as presented in Supplementary Table 7. For tariffs, production costs and border compliance costs, we evaluate the median value across all regions, and set all values above this value to the median value. While there are technical, social, political economy and natural constraints to reduce production costs, we assume that the median value globally is attainable across all production regions, for instance by closing the yield gap, automation and other efficiency gains, and improved land-use management. The cost difference between the existing and median value is the potential cost reduction that could be achieved. In regions where the cost component is less than the median value, no change is made. For the transport cost, we have to adopt a different approach, as the average transport cost depends on the distance travelled, with longer travel distances resulting in a lower average cost per tonne per kilometre. This is because, generally speaking, the cost to ship from port to hinterland are higher than the maritime transport costs. Moreover, longer-distance trade generally has a relatively larger share of the total transport costs allocated to maritime transport. To find a benchmark transport cost, we first find the average transport cost per tonne per kilometre (ATC in USD t^−1^ km^−1^) and fit a regression formulation based on the distance (*D*) between exporting (*e*) and importing (*i)* country pair, per mode of transport used (*m*, which is maritime, road or rail)

$$\mathrm{ln}\left({\mathrm{ATC}}_{e,i,m}\right)={\beta }_{0,m}+{\beta }_{1,m}\mathrm{ln}\left({D}_{e,i,m}\right)+{\epsilon }_{m}.$$

As such, for every pair of administrative regions, we evaluate whether the modelled ATC are above or below the regression based ATC, and set all transport costs above this value to the regression ATC under the assumption that this could be a realistically attainable ATC under investments in land, port and maritime infrastructure.

Alongside the cost reduction strategy, we cluster each administrative region based on the cost breakdown of four main cost groups that contribute to the landed costs (production, border, tariff and transport). On the basis of this breakdown, we cluster administrative regions into six distinct clusters using an unsupervised clustering algorithm (*k*-means). Six clusters was found to be the optimum cluster size, although in Supplementary Figs. 10–11, we also show the results for five and seven clusters.

### Reporting summary

Further information on research design is available in the Nature Portfolio Reporting Summary linked to this article.

## Supplementary information

Supplementary Information. Supplementary Methods 1–3, Figs. 1–12, Tables 1–8 and references.

Reporting Summary

## Data Availability

The production cost data are available via Zenodo at https://zenodo.org/record/7701784#.ZBMVXnaZOUk (ref. ^40^). The BACI harmonized trade dataset and the MacMap-HS6 applied tariff dataset are both available via the CEPII website (https://www.cepii.fr/CEPII/en/bdd_modele/bdd_modele.asp). The global gridded population dataset is available at https://earthdata.nasa.gov/data/catalog/sedac-ciesin-sedac-gpwv4-popdens-r11-4.11 and global subnational administrative boundaries are available via GADM (https://gadm.org). The global gridded production and yield data are available via MAPSPAM (https://mapspam.info/). The subnational transport cost dataset and model output to reproduce the analysis are available via Zenodo at https://zenodo.org/records/14028714 (ref. ^41^).

## Code availability

The code needed to analyse the data and reproduce the figures is available via Zenodo at https://zenodo.org/records/14028714 (ref. ^41^).
