# Supplementary material for: Heterogeneities in landed costs of traded grains and oilseeds contribute to unequal access to food
Source: Nat Food. 2025 Jan 6;6(1):36–46. doi: 10.1038/s43016-024-01087-7 (PMC11772242; doi:10.1038/s43016-024-01087-7)
Supplement: Supplementary file 1 — Supplementary Methods 1–3, Figs. 1–12, Tables 1–8 and references. [file 43016_2024_1087_MOESM1_ESM.pdf]

nature food

Supplementary information https://doi.org/10.1038/s43016-024-01087-7

Heterogeneities in landed costs of traded grains and oilseeds contribute to unequal access to food

In the format provided by the authors and unedited

Contains:
Supplementary Methods 1-3
Supplementary Figures 1-12
Supplementary Tables 1-8
Supplementary References

Supplementary Methods

Supplementary Methods 1: Scaling production cost function

The production cost database includes one value per crop and country (in USD/ha). In order to translate this to production cost in USD per tonnes, we divide by the regional (admin-average) yield. However, there are known differences in production cost between high yielding and low yield production systems and regions. As such, we add a correction factor that increases or decrease the regional production cost (in USD/t) depending on regional yield versus the country median yield (median across regions).

In order to characterize this relationship, we collect regional production data from the United States[1], India[2] and the EU[3]. Based on this data, we evaluate the relationship between the regional deviation from median yields and the production cost per hectare for different crops. Although the regions are relatively aggregated, for some crops we find that clear relationship. We adopt a relatively simple approach, that is consistent with our global modelling study, to create a generalisable cost correction factor (CCF) across countries and crops, using the following relationship:

$$CCF = 0.75 \; if \; yield_{region} < 0.5 \, yield_{median}$$
$$CCF = 1.25 \; if \; yield_{region} > 1.5 \, yield_{median}$$
$$CCF = 1 + 0.5 \, (yield_{region}/yield_{median} - 1) \; otherwise$$

Based on this, we can create regional production cost in USD/t using:

$$Production\ cost\ (USD/t) = \frac{Production\ cost\ (USD/ha) * CCF}{yield_{region}}$$

[1] Obtained from: https://www.ers.usda.gov/data-products/commodity-costs-and-returns/
[2] Obtained from: https://eands.dacnet.nic.in/Cost_of_Cultivation.htm
[3] Obtained from: https://agridata.ec.europa.eu/extensions/FADNPublicDatabase/FADNPublicDatabase.html

Supplementary Methods 2: Description global transport model components
Below, we briefly describe the data sources used to capture all transport-related time and cost to ship goods from origin to destination, in our case between admin regions globally. A summary of the data sources is included in Supplementary Table 5.

Transportation networks
We create a global physical transport network based on three transportation modes: rail transport, road transport and maritime transport. The choice for using these is that grains and oilseeds are primarily transported by these modes. However, in some countries (e.g., U.S.A.), inland water transport is a widely used transportation mode. However, global data on river ports and river section suitable for transportation is lacking and hence not included for simplicity here.

We extract global road and rail transport networks from OpenStreetMap, which include the geographical location and certain characteristics (e.g., speed, road pavement, etc.). For rail transport, this includes the rail lines as well as rail stations, which can serve as intermodal points connected by road transport.

The global maritime transport network, consisting of over 1400 ports and the maritime transport routes that connect them, is derived based on (Verschuur, Koks and Hall, 2022). Here the authors created a port-to-port transport network based on vessel movement data between 2019 and 2020, which include the type, capacity, carrying capacity (capacity times payload) of vessels between ports globally and their travel time.

Hinterland transport cost
We have collected road and rail transport cost data from various online sources, resulting in a total of 39 countries for road transport data and 37 countries for rail transport data. We first corrected all cost data for 2021 USD values. We then set-up a regression formulation to gap-fill transport data for the missing countries. After testing several formulations, we find the best fit for a formulation where transport cost (TC) is regressed against continent dummies and the infrastructure index of the Logistics Performance Index (LPI)[4]:

$$ln(TC) = \beta_0 + \beta_1 LPI_{infra} + \beta_2 {LPI_{infra}}^2 + CD$$

With CD a continent dummy. The LPI infrastructure index is a score from 1 to 5, indicating the quantity and quality of transport infrastructure in a respective country.

For the transport-specific value of time (VOT), we use the regression formulation as found in previous work (Binsuwadan *et al.*, 2022), who performed a meta-analysis to find a regression formulation of the value of freight time across countries. This final formulation they adopt is a regression based on a country's GDP per capita, and a mode-specific constant, which is low for sea and rail transport (lower value of time) and high for air and road transport (higher value of time). This VOT value per country is utilised to scale the transport time on a certain segment into a dollar value, alongside the transport cost along that segment. The combined value of travelling over a segment is used to find the lowest utility connection between two places, which is thus a combination of the lowest cost and fastest transport routes across the network.

[4] See https/lpi.worldbank.org

Maritime transport cost

Maritime transport cost are less country-specific, given the global character of maritime transport, but more route and cargo specific. We adopt a vessel and route specific transport costs based on three variables: the type of vessel connecting ports, its capacity, and the utilization of that vessel. The look at three vessel types separately: container vessels, general cargo vessels, and dry bulk vessels. These vessel types are responsible for transporting agricultural trade.

The transport costs per vessel type scales predominantly with the size of the vessel, expressed in terms of its deadweight tonnage (DWT), which reflect the economies of scale of transporting goods on larger vessels (which serve on busier, or higher volume, routes).

Based on work of (Bernacki, 2021a) and (Bernacki, 2021b), the following specifications are adopted:

$$TC_{drybulk} = 4.1DWT^{-0.75}$$

$$TC_{Generalcargo,container} = 0.65DWT^{-0.56}$$

However, in reality, vessels are not always full, and transport costs can differ for routes with large trade imbalances, with transport cost lower for fully loaded vessels than empty vessels. As such, instead of the DWT per route and vessel, we use the average carrying capacity of vessels on these routes, which is simply the maximum carrying capacity times the payload. This results in three networks of transport costs between ports that have a direct maritime connection.

Similar as with the road and rail transport, we use a VOT value from (Binsuwadan *et al.*, 2022), but for maritime transport.

Border and Custom compliance time and cost

Custom compliance time and cost include all the time and cost associated with clearance and inspiration required at the border. These cost can vary across country pairs, and the mode of transport taken, primarily whether land transport is used or whether a port or airport is used. We extract the custom compliance time and cost between country pairs from the 'Doing Business' database from the World Bank (which will soon be replaced by the B-READY imitative). To scale this across country pairs, we utilize a regression formulation that captures the customs time (CT) and cost (CC) based on the Customs efficiency indicator of the Logistics Performance Index:

$$ln(CT_{port}) = \beta_0 + \beta_1 LPI_{custom,O} + \beta_2 LPI_{custom_D}$$
$$ln(CC_{port}) = \beta_0 + \beta_1 LPI_{custom,O} + \beta_2 LPI_{custom_D}$$

With O being the exporting (origin) country and D being the importing (destination country). These custom compliance costs are only associated with the port, as we will add a separate dwell time estimate + handling to the ports (see below). The total customs time and cost per country pair are the sum of the CT and CC of the origin country and the destination country.

For land border crossings, we capture the border and custom compliance time and cost into a single indicators: the border dwell time (DT) and border handling cost (HC). The border dwell time and handling costs, include all time and cost associated with compliance with a country's

custom regulations and inspections at the land border. We run a similar regression but only when goods leave the country and cross the first land border:

$$ln(DT_{land}) = \beta_0 + \beta_1 LPI_{custom,O}$$
$$ln(HC_{land}) = \beta_0 + \beta_1 LPI_{custom,O}$$

The dwell time and handling cost are added all land-crossings in the dataset, such that the total dwell time and border compliance costs depend on the number of borders that have to be crossed to trade from origin to destination country.

The border and custom compliance cost are irrespective of the size of the shipment. We simplicity, we assume that these costs are associated with the shipment of a single truck with a payload of 25 tonnes, which allows us to convert the border and custom compliance cost (which are in USD) to a suitable unit for analysis (USD/t).

Port costs and time

On top of the custom compliance time and cost at ports, we also add a vessel turnaround time, port dwell time, port handling cost to every port. The vessel turnaround time captures the time it takes for vessels to enter a port, unload the cargo and leave the port boundary. The can differ substantially from port-to-port, capturing the efficiency of port operations. Based on information in (Verschuur, Koks and Hall, 2022), we adopt vessel type and port specific average turnaround time values at every port.

The port handling cost captures the costs that have to be paid to the terminal operators for handling goods, either for transhipment purposes or import and export through the hinterland. The dwell time is the average time cargo spends in the port storage facility before it leaves the port premises (either loaded on a vessels, or loaded on trucks or rail). For dry bulk and general cargo vessels, we adopt generic values across ports, given limited information how these cost and time differ between ports. For container terminals, this information is available. We have collected a database the handling cost of a container (in US per TEU) across 237 ports. We translated this handling cost value into a regional cost estimate and assigned this to each port in that region. Similar, we collected container dwell times at over 800 ports globally from various sources. Again, we find a regional average container dwell time and assign this to ports in these various regions we adopted (22 regions).

Using this information, we have port specific information on the time and cost associated with shipping goods through specific ports. The total dwell time through ports is found by adding the vessel turnaround time, the cargo dwell time and the custom compliance time. Similarly, the total port and border handling cost includes the port terminal charge and the custom compliance costs.

Hinterland switching costs

We also include cost and time values to move goods from storage facilities to trucks/rail, from trucks to final customers, from trucks to rail, and from trucks/rail to ports. All values are similar across countries and adopted based on data in (Verschuur, Koks and Hall, 2022).

Global transport cost estimates

With all cost and time estimates added to the different network components, we can derive admin-to-admin (based on admin centroids) shortest path estimates using the different modes (road, rail, dry bulk, general cargo, container) using the Dijkstra's shortest path algorithm

(Dijkstra, 1959), implemented in the iGraph python package. This allows us to find the highest utility (characterised by the generalized cost, GN, function) path route between admin-pairs, and time and transport cost associated with this lowest GN path.

Supplementary Methods 3: Radiation model versus other trade allocation models
The radiation model that is introduced in this paper is less frequently applied to downscale trade flows from a country-to-country level to an administrative region-by-region level. More conventional, simpler, methods to do so are by using ‘proportionate scaling’ or using a (uncalibrated) gravity model.

The proportionate scaling method simply assumes that the trade flow can be scaled based on the relatively supply and demand proxies. For instance, using production (*P*) and demand (*D*) as supply and demand proxies, the trade flow between two administrative regions (*importing, ir, and exporting, er*) belong to the set of administrative regions in the origin (*ec*) and importing countries (*ic*) is:

$$T_{er,ir,g} = T_{ec,ic} * \frac{C_{er,g} * D_{ir,g}}{\sum_{er,ir} C_{er,g} * D_{ir,g}}$$

Here, only the production and demand information is used, and no information on the distance or costs to source between any two administrative regions. Hence, the distribution of origin flows will not differ across sourcing countries and only be determined by the location of production.

A gravity model will also take the distance *(d*) between regions into consideration. However, gravity models calibrated on bilateral trade data cannot simply be applied to model subnational trade flows. Hence, the simplest way to account for distance is using a linear scaling approach:

$$T_{er,ir,g} = T_{ec,ic} * \frac{(C_{er,g} * D_{ir,g})/d_{ir,er,g}}{\sum_{er,ir}(C_{er,g} * D_{ir,g})/d_{ir,er,g}}$$

However, differences in distance may not necessarily be a good proxy for the differences in sourcing costs, given the cost variations between hinterland transport and maritime transport costs. Moreover, differences in transport costs could be compensated for having lower production costs (given higher yields).

The elegance of the radiation model is that it bring together information on production, demand, yields (which determine production costs) and trade costs within one formulation. The rationale here being that trade will increase between any exporting and importing region for which it is cheap to source from (in terms of the landed costs), but influenced by the amount of production in the exporting regions that have an even lower sourcing costs. Compared to the proportionate allocation method, the distribution of origin flows will differ across sourcing countries.

For illustrative purposes, Supplementary Figure 12 shows the distribution of origin flows of soybean exports from Brazil to respectively China, Great Britain, Bolivia, Argentina and the United States, with ‘Base’ representing the location of soybean production. It similarly shows the origin of flows of maize exports from the United States to respectively China, Columbia, Canada, Mexico and Saudi Arabia. Moreover, in Supplementary Table 8, we compare the modelled landed costs for these three methods. The table shows that, generally speaking, the radiation model will cause lower landed costs compared to the other method, given the preference to source commodities from production regions that have lower transport costs. In particular for geographical larger and more dispersed countries, with multiple production locations across the country, the differences between the methods will be larger.

While we believe the radiation model is a preferred trade flow disaggregation method, it should be interpreted with care as other, non-trade cost factors, determine the locations from which commodities are sourced. Moreover, more research into validating subnational trade flow methods is required, which is limited given the lack of detailed validation data on this geographically refined scale.

Supplementary Figures

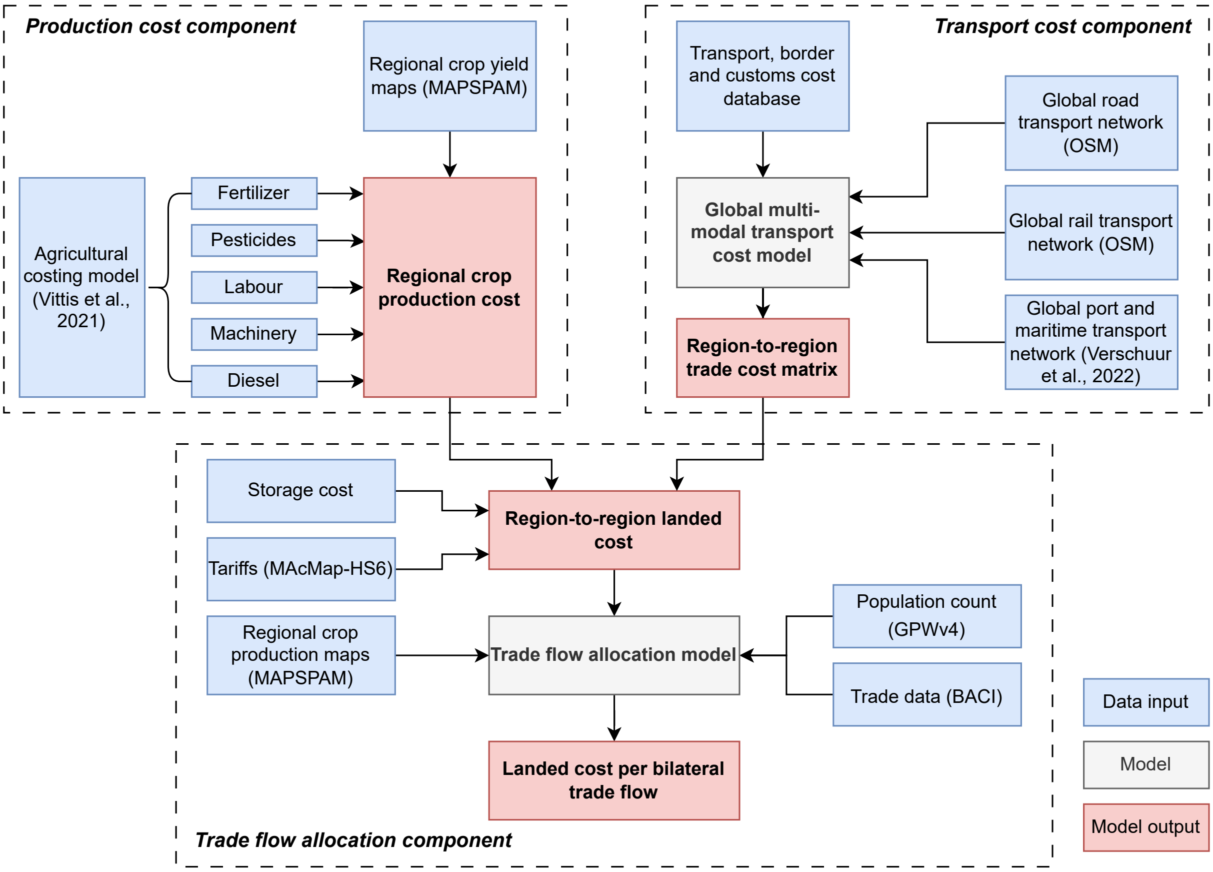

Supplementary Figure 1. Workflow, including data input, model components and data output.

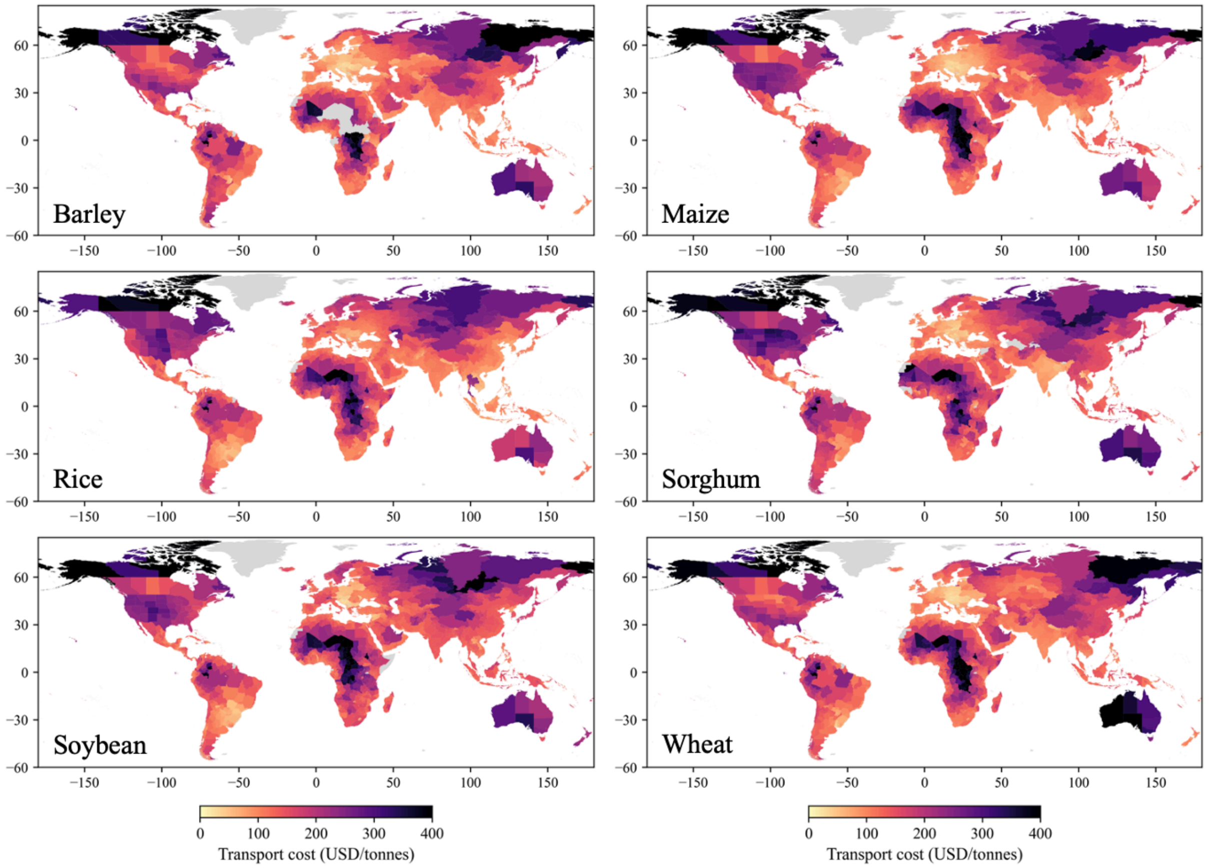

Supplementary Figure 2. Weighted average transport cost from field to customer. Basemap is from GADM (gadm.org).

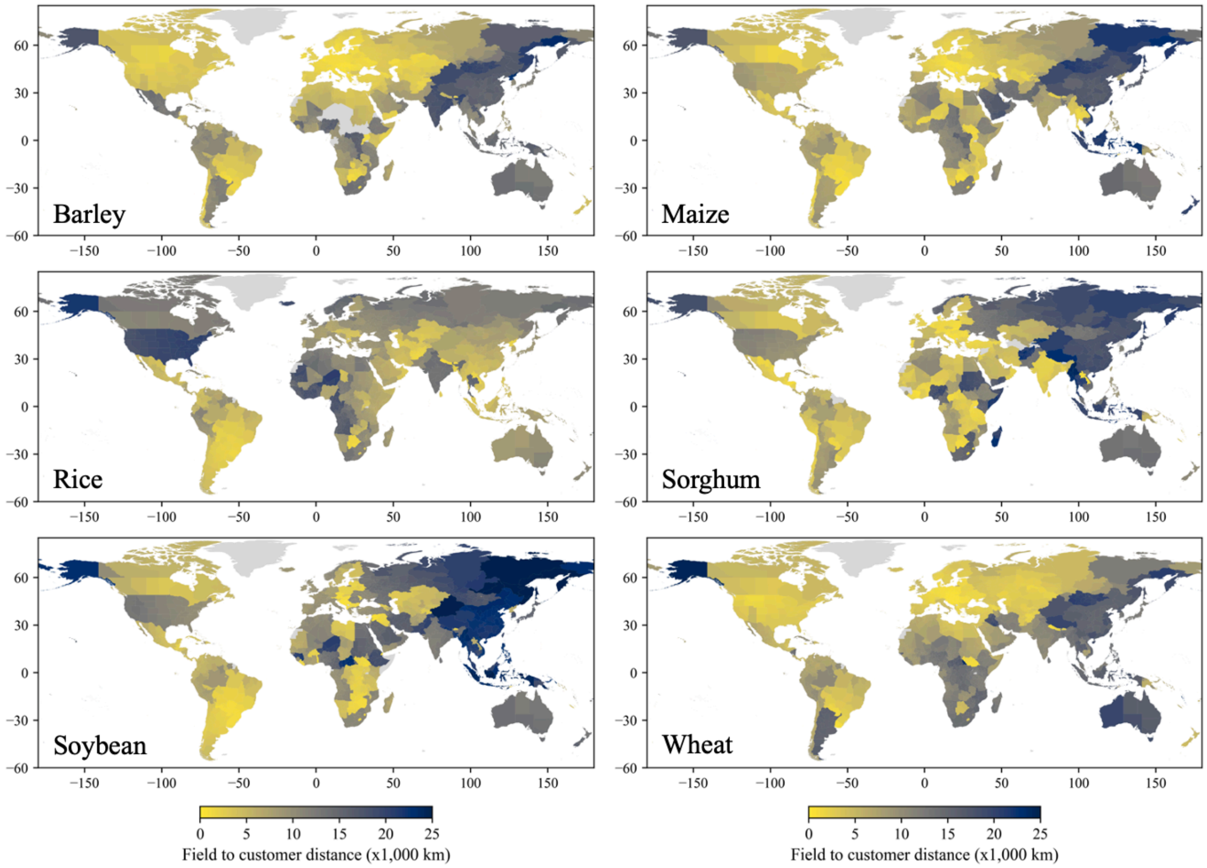

Supplementary Figure 3. Weighted average field-to-customer distance per crop type. Basemap is from GADM (gadm.org).

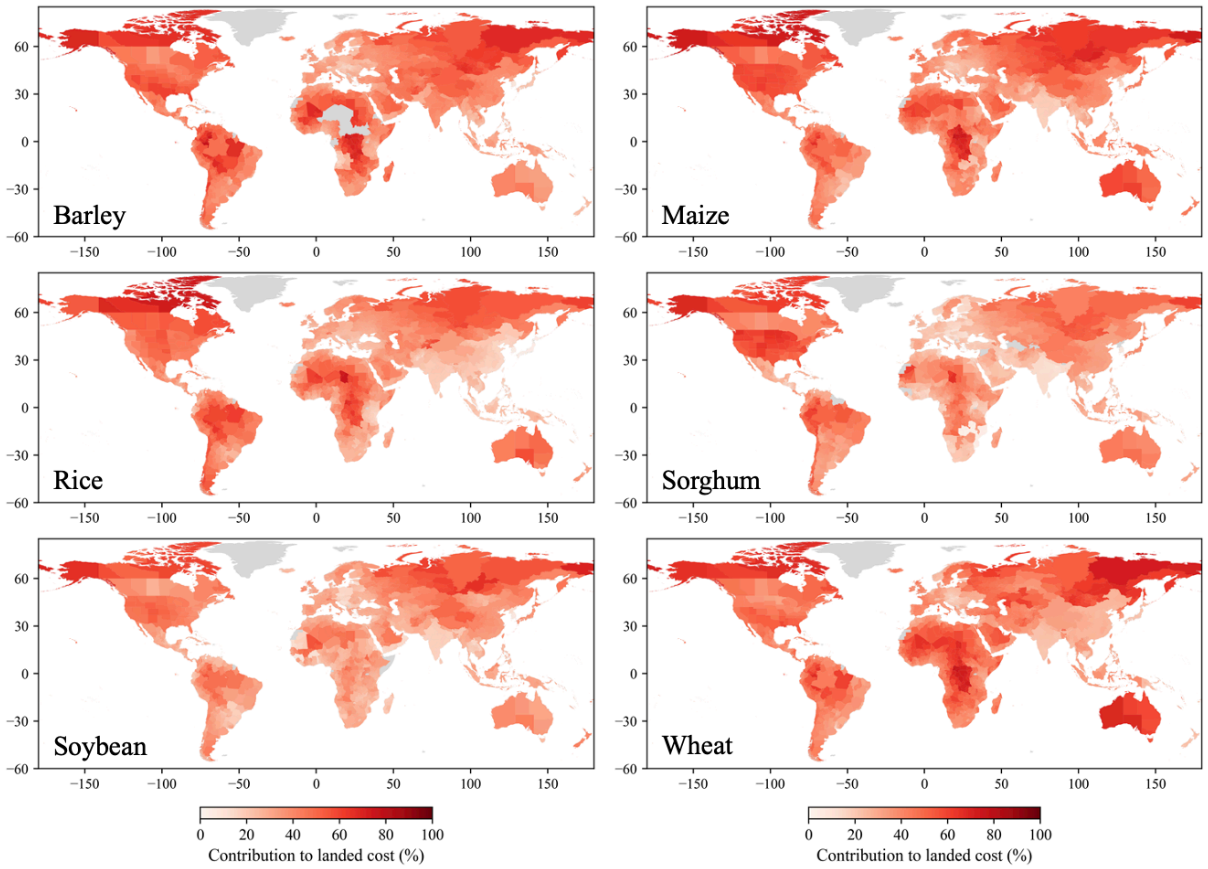

Supplementary Figure 4. The share of transport cost to landed cost. Basemap is from GADM (gadm.org).

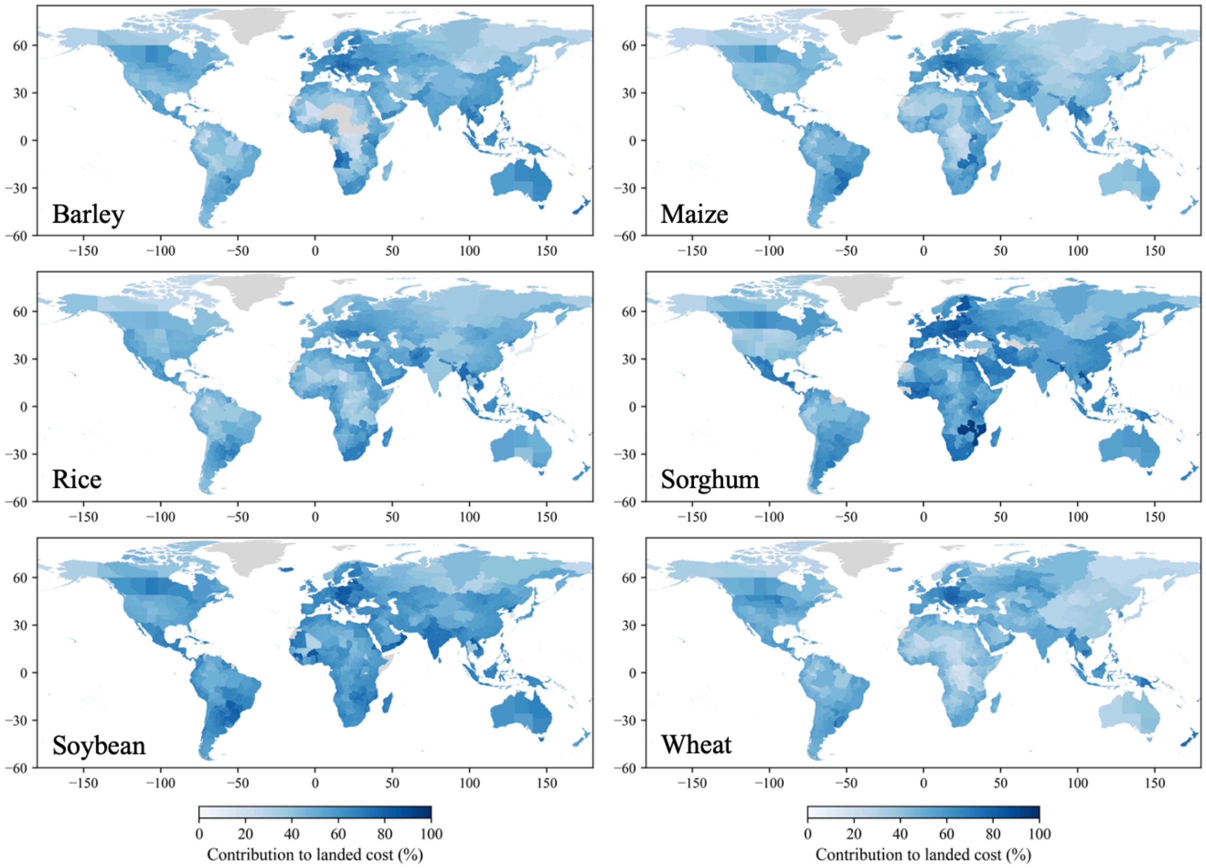

Supplementary Figure 5. The share of production plus storage cost to landed cost. Basemap is from GADM (gadm.org).

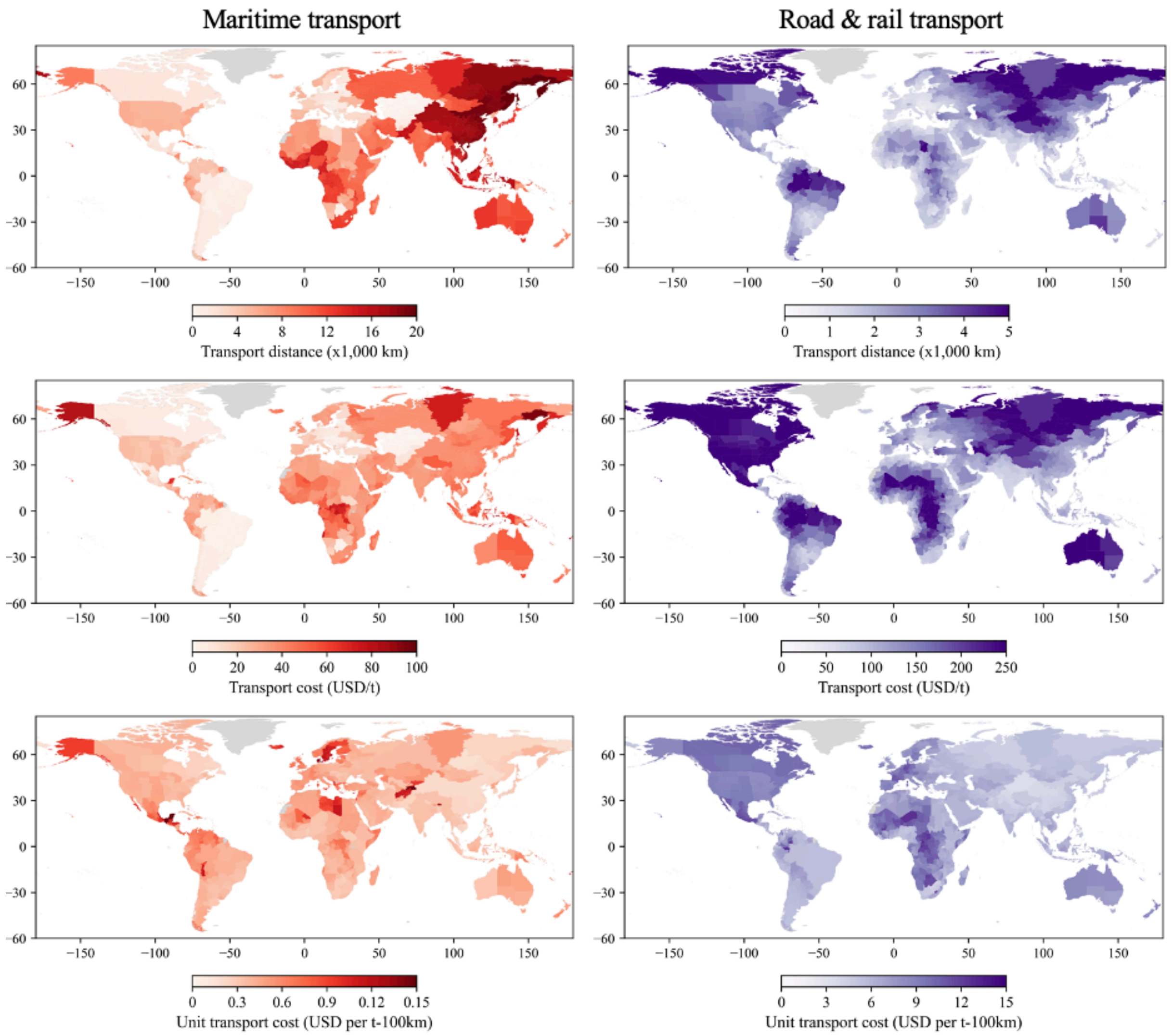

Supplementary Figure 6. Breakdown of global (trade) weighted average transport cost. Breakdown of the weighted average transport cost, weighted by the quantity of trade and across all crops, in a maritime transport cost (top row) and distance (middle row) and a road and rail cost and distance. The bottom row illustrates the unit transport costs per tonne per 100 kilometre of transport. Note the differences in scale between the two figures per row. Road & rail transport here refers to both the road and rail transport cost to reach ports as well as for land-based transport cost to trade via land-connections. Basemap is from GADM (gadm.org).

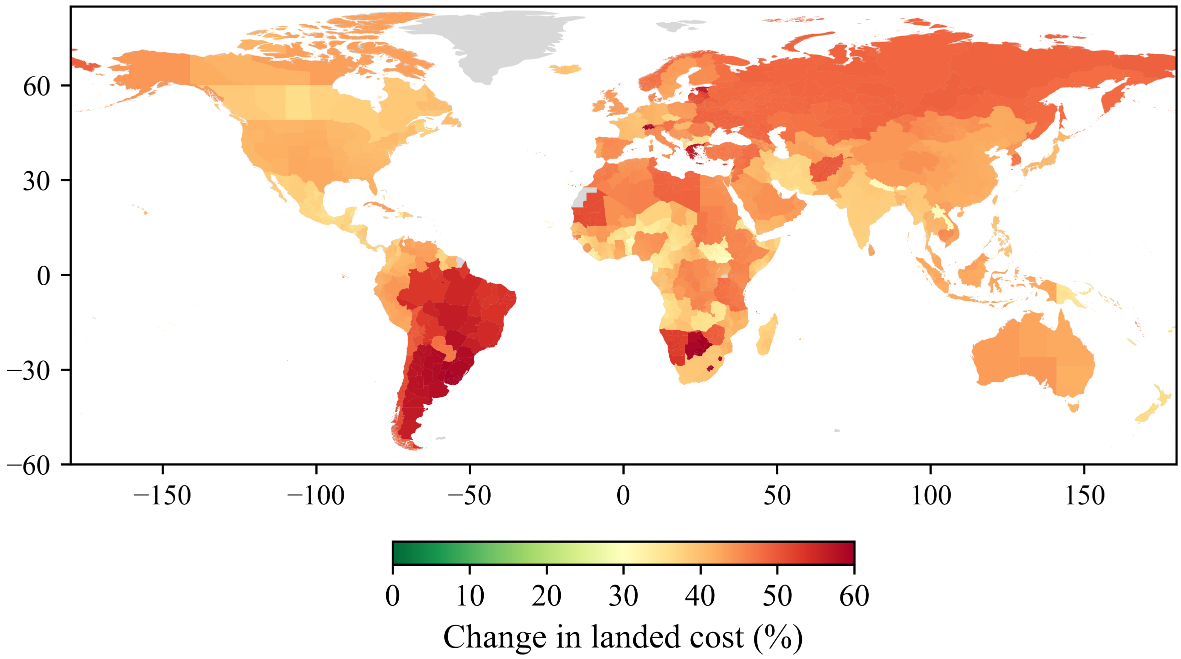

Supplementary Figure 7. Relative change in landed cost across the crop types for the price shock calculated. Basemap is from GADM (gadm.org).

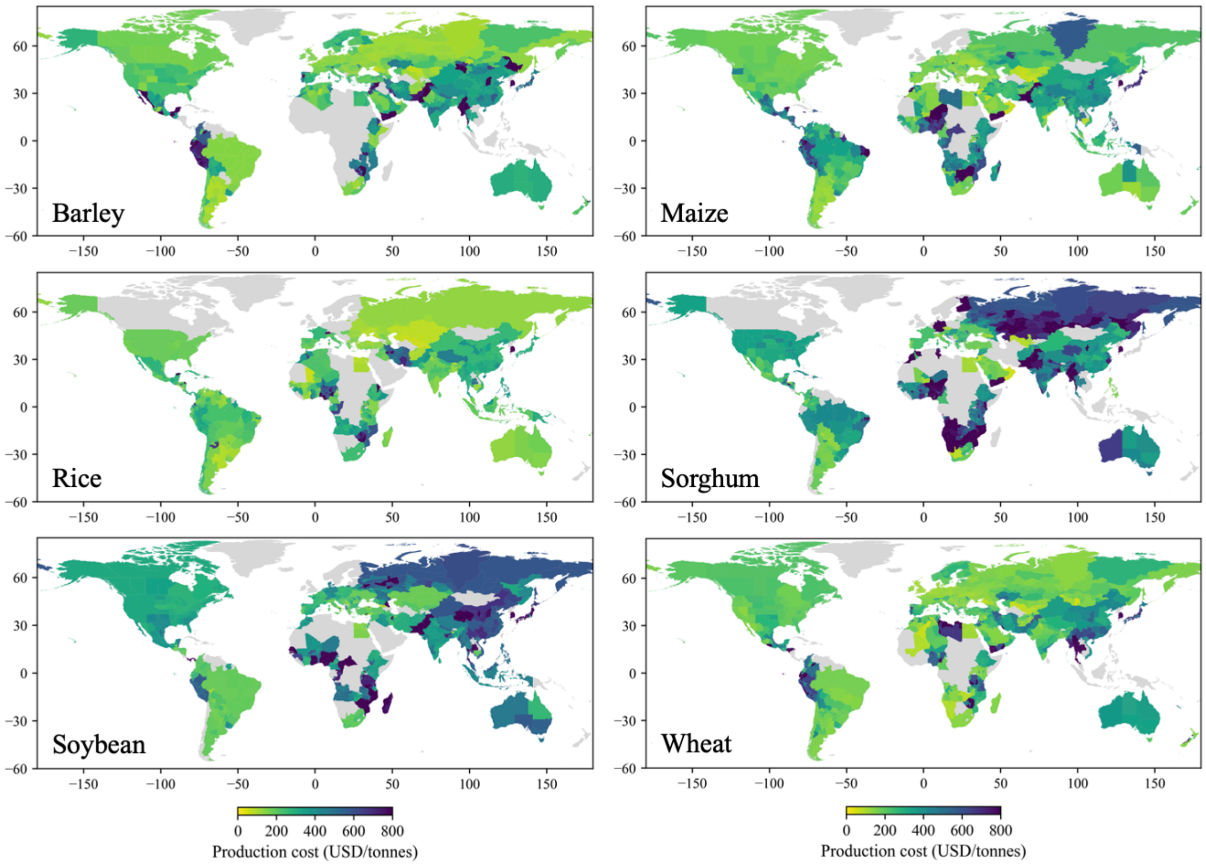

Supplementary Figure 8. Regional production cost of crop exporting regions in USD/t. Basemap is from GADM (gadm.org).

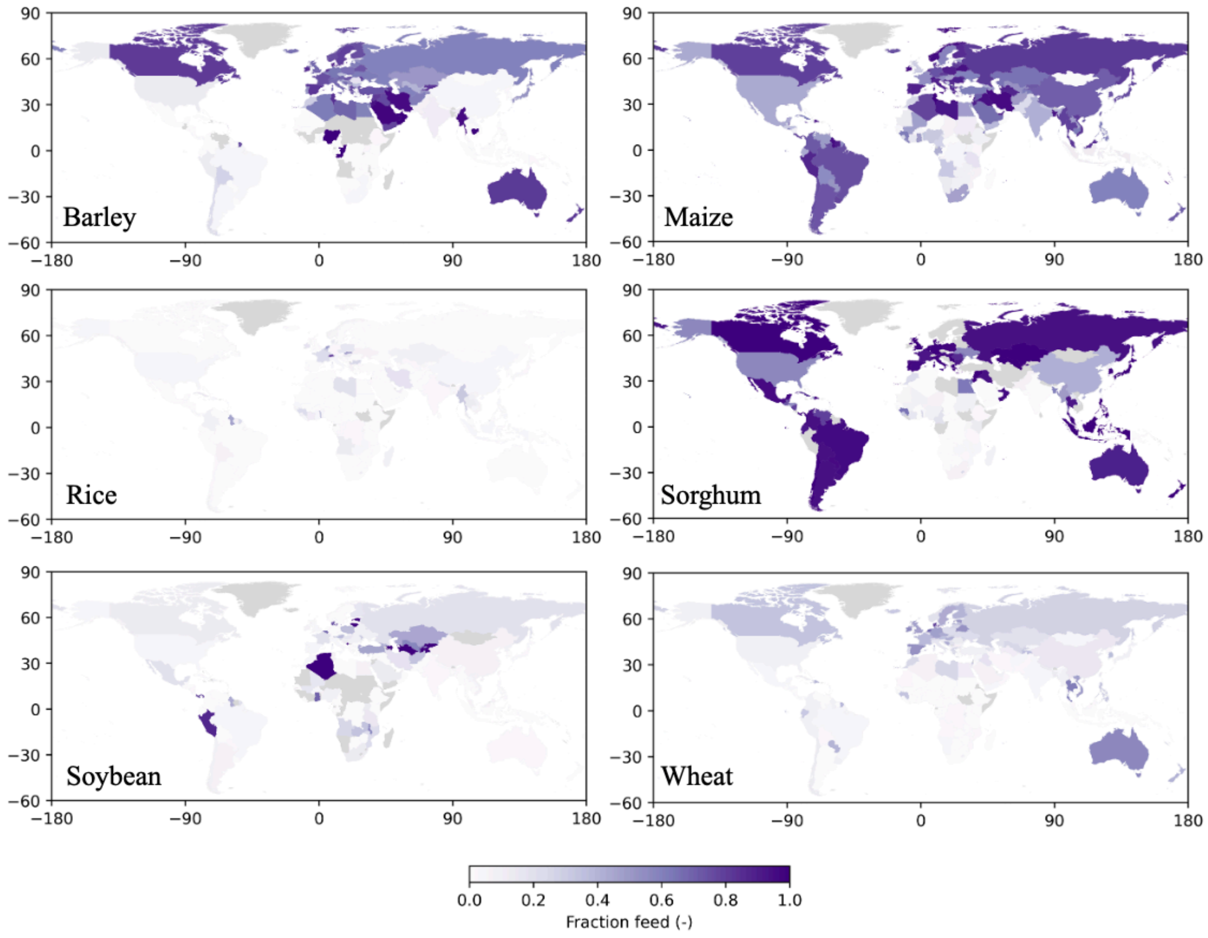

Supplementary Figure 9. Share of feed demand in total grain demand. The share of consumption for feed purposes over all human and non-human grain consumption. Data based on the FAO Food Balance Tables, averaged over the period 2016-2019.

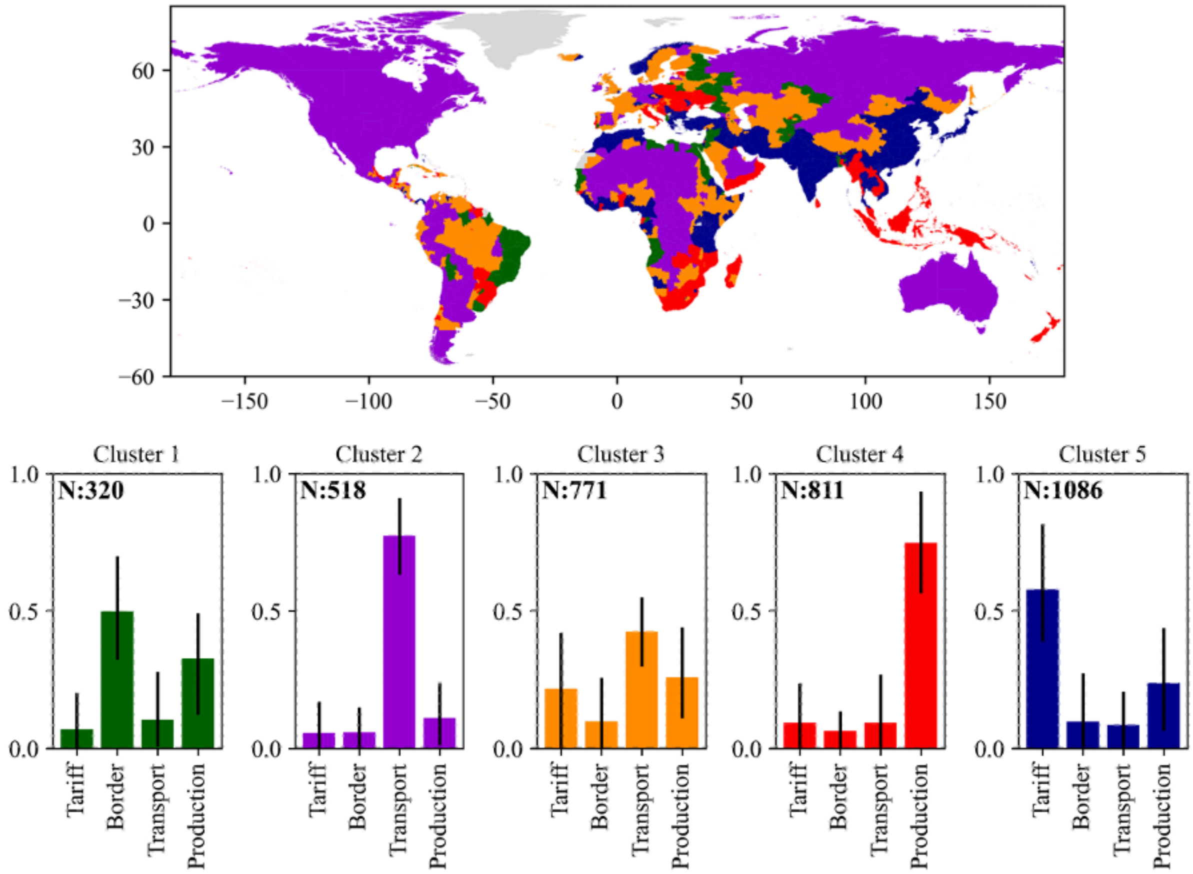

Supplementary Figure 10. Cluster results of Figure 5 for five clusters. For each cluster, the N indicates the number of subnational administrative regions that fall within this cluster. The bar indicates the median value across the administrative regions within a cluster, whereas the error bars indicate the 10th and 90th percentile range of the administrative regions within a cluster.

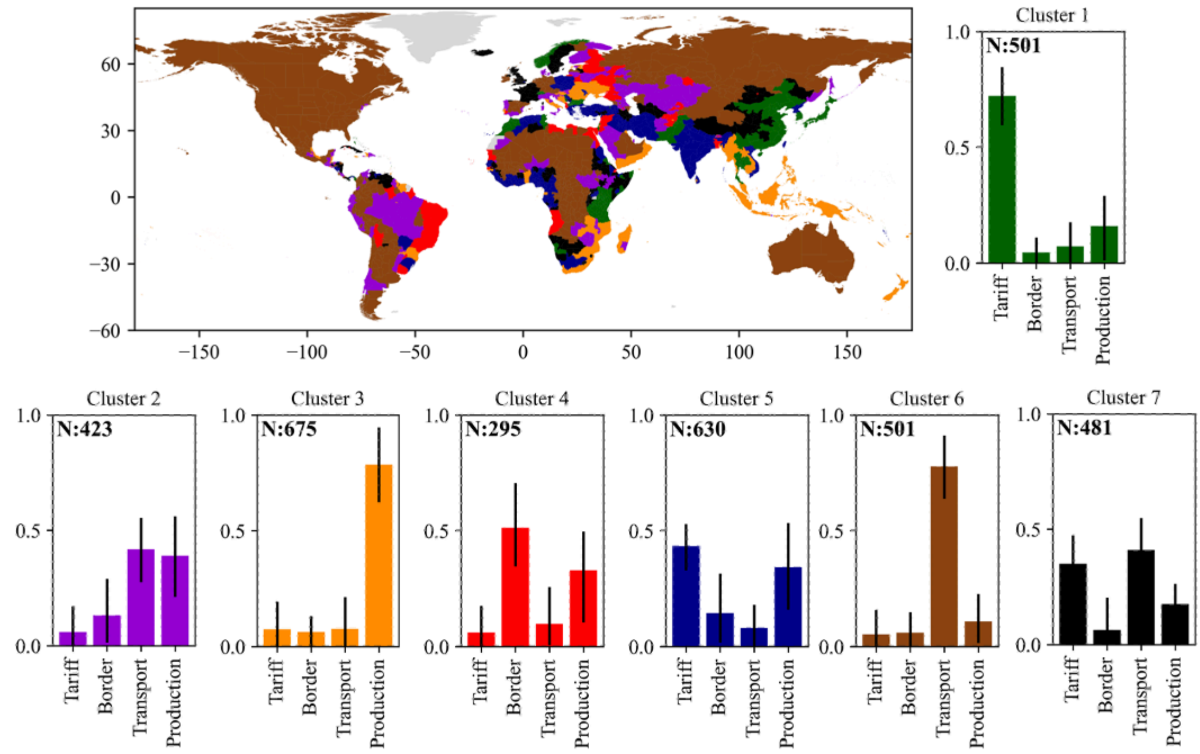

Supplementary Figure 11. Cluster results of Figure 5 for seven clusters. For each cluster, the N indicates the number of subnational administrative regions that fall within this cluster. The bar indicates the median value across the administrative regions within a cluster, whereas the error bars indicate the 10th and 90th percentile range of the administrative regions within a cluster.

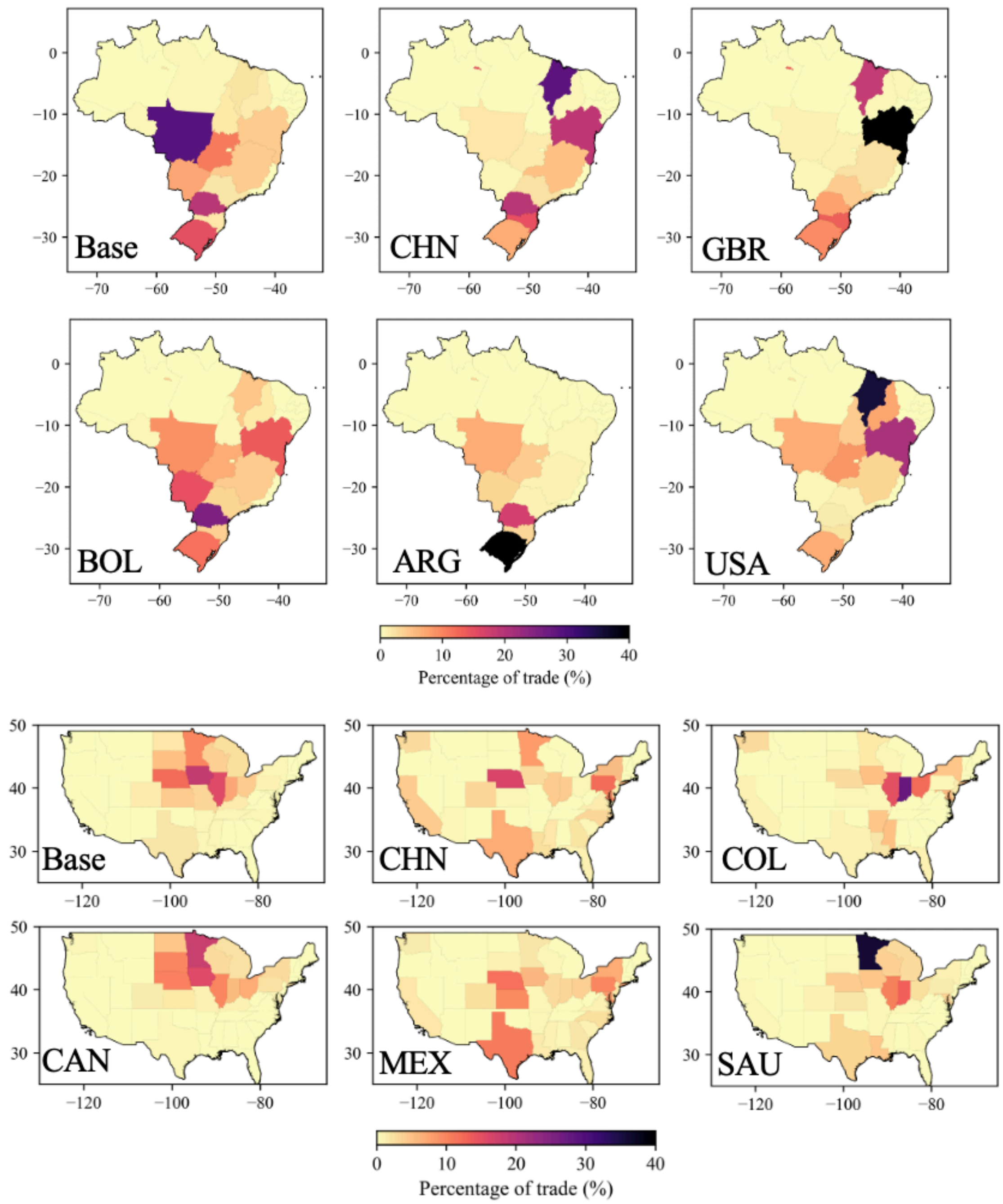

Supplementary Figure 12. Origin of commodity trade flows for the radiation model. The top figures show the distribution of origin trade flows (exporting administrative regions) for soybean exports from Brazil, and how it differs per importing country: China (CHN), Great Britain (GBR), Bolivia (BOL), Argentina (ARG) and the United States (USA). The Base model shows the origin using the Proportionate allocation method, which does not vary across importing country. The bottom set of figures shows the same result but for United States maize exports for five major imports: China (CHN), Columbia (COL), Canada (CAN), Mexico (MEX) and Saudi Arabia (SAU).

Supplementary Table 1. Key summary statistics of crops considered. Production quantity, feed, share consumed kcal, share consumed quantity all come from the FAO Food Balance data (average across last 5 available years), whereas the trade data comes from the BACI trade database (2017-2021 average)

**Crop**	**Production (billion t)**	**Food (billion t)**	**Feed (billion t)**	**Share kcal consumed (%)**	**Share quantity consumed (%)**	**Trade (billion t)**
Barley	0.15	0.01	0.09	0.26	0.14	0.04
Maize	1.14	0.14	0.63	5.34	2.67	0.19
Rice	0.75	0.59	0.03	18.14	11.25	0.05
Sorghum	0.06	0.03	0.02	0.99	0.5	0.01
Soybeans	0.35	0.01	0.02	0.44	0.19	0.16
Wheat	0.76	0.50	0.13	18.5	9.49	0.22
Total	3.21	1.28	0.92	43.67	24.24	0.67

Supplementary Table 2. Regional differences in weighted landed cost, including the 10th, 50th and 90th percentile.

Region	Crop	10th	50th	90th
South Asia	BARL	205.5	254.0	369.3
South Asia	MAIZ	217.9	316.3	417.9
South Asia	RICE	193.3	239.3	384.5
South Asia	SORG	279.9	427.5	592.9
South Asia	WHEA	192.6	247.1	426.3
South Asia	SOYB	319.4	406.7	531.7
Sub-Saharan Africa	BARL	190.6	297.7	494.1
Sub-Saharan Africa	MAIZ	212.2	287.3	490.0
Sub-Saharan Africa	RICE	242.4	370.2	510.7
Sub-Saharan Africa	SORG	381.4	498.8	750.1
Sub-Saharan Africa	WHEA	209.0	295.0	425.3
Sub-Saharan Africa	SOYB	317.0	440.9	643.4
Western Asia	BARL	191.9	266.6	443.7
Western Asia	MAIZ	191.3	269.1	416.5
Western Asia	RICE	220.9	284.0	412.3
Western Asia	SORG	275.5	394.8	600.2
Western Asia	WHEA	164.9	233.4	465.8
Western Asia	SOYB	266.3	306.8	474.0
Latin America & Caribbean	BARL	187.2	227.1	394.3
Latin America & Caribbean	MAIZ	231.2	287.4	337.1
Latin America & Caribbean	RICE	206.5	278.5	341.6
Latin America & Caribbean	SORG	357.4	403.6	438.4
Latin America & Caribbean	WHEA	232.6	296.3	409.9
Latin America & Caribbean	SOYB	268.8	381.7	436.7
Oceania	BARL	360.3	383.2	503.3
Oceania	MAIZ	262.9	295.1	339.0
Oceania	RICE	278.8	358.0	496.7
Oceania	SORG	410.3	475.2	513.8
Oceania	WHEA	411.5	449.1	555.3
Oceania	SOYB	419.1	541.8	815.0
North America	BARL	290.7	360.1	431.6
North America	MAIZ	292.5	338.3	436.1
North America	RICE	324.3	444.1	552.1
North America	SORG	350.3	422.6	521.3
North America	WHEA	307.8	382.1	447.5
North America	SOYB	359.4	451.9	562.4
North-West Europe	BARL	165.5	195.2	260.8
North-West Europe	MAIZ	173.8	219.9	318.2
North-West Europe	RICE	249.8	369.8	624.6
North-West Europe	SORG	264.7	304.9	380.9

North-West Europe	WHEA	153.2	184.4	298.2
North-West Europe	SOYB	290.4	393.1	488.3
North Africa	BARL	200.9	232.2	297.2
North Africa	MAIZ	187.5	230.5	314.9
North Africa	RICE	224.3	335.8	408.7
North Africa	SORG	426.9	472.8	554.1
North Africa	WHEA	165.9	216.7	334.5
North Africa	SOYB	279.3	380.6	411.4
East Asia	BARL	220.7	363.0	541.0
East Asia	MAIZ	251.3	331.6	1129.5
East Asia	RICE	347.5	473.2	2703.3
East Asia	SORG	314.5	454.1	497.5
East Asia	WHEA	295.3	531.6	750.0
East Asia	SOYB	295.7	321.2	461.6
South-East Asia	BARL	295.7	377.6	397.9
South-East Asia	MAIZ	212.7	254.8	342.7
South-East Asia	RICE	238.0	349.6	435.2
South-East Asia	SORG	305.4	454.1	501.1
South-East Asia	WHEA	198.9	288.5	442.5
South-East Asia	SOYB	334.3	437.1	718.5
Eastern Europe	BARL	151.1	179.8	263.0
Eastern Europe	MAIZ	146.2	165.4	214.5
Eastern Europe	RICE	205.5	293.4	519.0
Eastern Europe	SORG	236.1	344.6	386.8
Eastern Europe	WHEA	151.4	173.7	278.2
Eastern Europe	SOYB	230.4	353.0	473.3
Central Asia	BARL	126.9	209.2	316.0
Central Asia	MAIZ	126.6	145.7	301.3
Central Asia	RICE	207.4	497.1	619.8
Central Asia	SORG	234.3	342.2	737.1
Central Asia	WHEA	196.8	322.1	365.2
Central Asia	SOYB	271.4	393.7	615.2
Southern Europe	BARL	176.7	223.6	311.3
Southern Europe	MAIZ	177.8	230.3	326.9
Southern Europe	RICE	199.6	293.1	562.9
Southern Europe	SORG	282.5	381.2	483.6
Southern Europe	WHEA	176.7	224.4	349.5
Southern Europe	SOYB	282.1	337.7	436.5

Supplementary Table 3. Regional inequality in landed cost.

Region	Barley	Maize	Rice	Sorghum	Soybean	Wheat
Global	0.96	0.64	0.89	0.43	0.53	1.12
Central Asia	0.90	1.20	0.83	1.47	0.87	0.52
East Asia	0.88	2.65	4.98	0.40	0.52	0.86
Eastern Europe	0.62	0.41	1.07	0.44	0.69	0.73
Latin America & Caribbean	0.91	0.37	0.48	0.20	0.44	0.60
North Africa	0.41	0.55	0.55	0.27	0.35	0.78
North America	0.39	0.42	0.51	0.40	0.45	0.37
North-West Europe	0.49	0.66	1.01	0.38	0.50	0.79
Oceania	0.37	0.26	0.61	0.22	0.73	0.32
South Asia	0.65	0.63	0.80	0.73	0.52	0.95
South-East Asia	0.27	0.51	0.56	0.43	0.88	0.84
Southern Europe	0.60	0.65	1.24	0.53	0.46	0.77
Sub-Saharan Africa	1.02	0.97	0.72	0.74	0.74	0.73
Western Asia	0.94	0.84	0.67	0.82	0.68	1.29

Supplementary Table 4. Overview of transport network data used.

**Transport mode**	**Subcategory**	**Data**
Road	Highway, trunk, primary, secondary, tertiary	OpenStreetMap
Rail		OpenStreetMap
Maritime	Container, dry bulk, general cargo	Maritime transport connections per vessel type between 1400 ports for period 2019-2020, including the capacity of vessels. For details, see Verschuur et al. (2022).

Supplementary Table 5. Overview of data sources and extrapolation method used per transport friction consider.

**Transport friction**	**Unit**	**Database**	**Extrapolation method**
Road transport costs	USD per tonnes-km	Database created based on literature review (39 countries)	Regression formulation based logistics performance infrastructure indicator and continent dummy.
Rail transport costs	USD per tonnes-km	Database created based on literature review (37 countries)	Regression formulation based logistics performance infrastructure indicator and continent dummy.
Maritime transport cost	USD per tonnes-km	Based on regression formulation in Bernacki et al. (2021)	Transport costs assigned per route based on the regression formulation and the average size of the vessel travelling on a specific route.
Value of freight travel time	USD per tonnes-h	Based on regression formulation in Binsuwadan et al. (2022)	Added to every country and applied for every trade connection based on value of the importing country.
Port handling cost	USD per tonnes	Database created based on literature review.	Different port handling costs per vessel type.
Port dwell time	Hours	Database created based on literature review	Extrapolation based on regional average estimates.
Vessel turnaround time	Hours	Median turnaround time per vessel and port as derived in Verschuur et al. (2022).	Port and vessel specific information.
Customs compliance costs ports	USD per tonnes	Doing Business report and database	Regression formulation based on origin and destination logistics performance customs indicator. A payload of 25 tonnes is assumed.
Border dwell time	Hours	Doing Business report and database	Regression formulation based on origin logistics performance customs indicator.
Border costs	USD per tonnes	Doing Business report and database	Regression formulation based on origin logistics performance customs indicator. A payload of 25 tonnes is assumed.
(Un)Loading costs	USD per tonnes	Different sources as summarised in Verschuur et al. (2022)	Different values for:<br>-origin/destination centroid to road network;<br>-road to rail network;<br>-road to port;

			-rail to port.
(Un)Loading time	Hours	Different sources as summarised in Verschuur et al. (2022)	Different values for:<br>-origin/destination centroid to road network;<br>-road to rail network;<br>-road to port;<br>-rail to port.

Supplementary Table 6. Overview of price shock assumptions

**Cost component**	**Change**	**Source**
Pesticides	+200%	World Bank Commodity Market Outlook
Fertilizer	+200%	World Bank Commodity Market Outlook
Diesel for farm machinery	+80%	Based on diesel price increase experienced in the United States based on data from the U.S. Energy Information Administration.
Transport	+50%	Based on the Grains and Oilseeds Freight Index from the International Grains Council (IGC).

Supplementary Table 7. Overview of cost reduction strategy

Cost component	Overview
Tariffs	All pairs of administrative regions where tariffs are >50th percentile are assumed to be reduced to the 50th percentile. This would reflect tariff reforms to reduce imports tariffs.
Border compliance	All pairs of administrative regions where border compliance are >50th percentile are assumed to be reduced to the 50th percentile. This would reflect trade facilitation strategies that reduce border and custom compliance costs, for instance through digitalization and other efficiency improvements.
Production	All exporting administrative regions where production costs (in USD/t) are >50th percentile are assumed to be reduced to the 50th percentile, which reflect investments to improve agricultural practises. This could be through more efficient land-use management practises, automatization and efficiency improvements.
Transport	Transport costs are first expressed as a USD/t/km per mode of transport (rail, road, maritime) and fit to a linear regression formulation (see Supplementary Methods). All transport cost in terms of USD/t/km which are above the fitted regression formulation are set to the regression formulation, which reflect investment to reduce transport costs, including investments in hinterland transport costs, port efficiency, and maritime connectivity.

Supplementary Table 8. The estimated landed cost between selected country pairs based on three types of trade disaggregation methods; proportionate allocation, (uncalibrated) gravity model, and the proposed radiation model in this study.

Origin	Destination	Commodity	Landed cost proportionate (USD/t)	Landed cost gravity (USD/t)	Landed cost radiation (USD/t)
Brazil	China	Soybeans	334.7	333.9	301.5
Brazil	Great Britain	Soybeans	347.8	347.7	284.9
Brazil	Bolivia	Soybeans	376.4	373.5	351.2
Brazil	Argentina	Soybeans	337.9	318.8	299.8
Brazil	United States	Soybeans	478.1	470.3	379.9
United States	China	Maize	371.9	407.8	371.9
United States	Columbia	Maize	324.2	321.5	306.3
United States	Canada	Maize	475.7	437.9	417.4
United States	Mexico	Maize	460.3	455.1	377.3
United States	Saudi Arabia	Maize	350.4	348.9	326.6

Supplementary References

- Bernacki, D. (2021a) 'Assessing the link between vessel size and maritime supply chain sustainable performance', *Energies*, 14(11). doi: 10.3390/en14112979.
- Bernacki, D. (2021b) 'Revealing the Impact of Increased Tanker Size on Shipping Costs', *European Research Studies Journal*, XXIV(Issue 1), pp. 604–621. doi: 10.35808/ersj/1983.
- Binsuwadan, J. *et al.* (2022) 'The value of travel time savings in freight transport: a meta-analysis', *Transportation*. Springer US, 49(4), pp. 1183–1209. doi: 10.1007/s11116-021-10207-2.
- Dijkstra, E. W. (1959) 'A Note on Two Problems in Connexion with Graph', *Numerische Mathematik*, 271, pp. 269–271.
- Verschuur, J., Koks, E. E. and Hall, J. W. (2022) 'Ports' criticality in international trade and global supply-chains', *Nature Communications*, 13(1), p. 4351. doi: 10.1038/s41467-022-32070-0.
