# Supplementary material for: Heterogeneities in landed costs of traded grains and oilseeds contribute to unequal access to food
Source: Nat Food. 2025 Jan 6;6(1):36–46. doi: 10.1038/s43016-024-01087-7 (PMC11772242; doi:10.1038/s43016-024-01087-7)
Supplement: Supplementary file 2 — Reporting Summary [file 43016_2024_1087_MOESM2_ESM.pdf]

nature portfolio

Corresponding author(s): Jasper Verschuur

Last updated by author(s): Nov 2, 2024

Reporting Summary

Nature Portfolio wishes to improve the reproducibility of the work that we publish. This form provides structure for consistency and transparency in reporting. For further information on Nature Portfolio policies, see our Editorial Policies and the Editorial Policy Checklist.

Statistics

For all statistical analyses, confirm that the following items are present in the figure legend, table legend, main text, or Methods section.

n/a	Confirmed	
☐	☒	The exact sample size (*n*) for each experimental group/condition, given as a discrete number and unit of measurement
☒	☐	A statement on whether measurements were taken from distinct samples or whether the same sample was measured repeatedly
☒	☐	The statistical test(s) used AND whether they are one- or two-sided<br>*Only common tests should be described solely by name; describe more complex techniques in the Methods section.*
☒	☐	A description of all covariates tested
☒	☐	A description of any assumptions or corrections, such as tests of normality and adjustment for multiple comparisons
☐	☒	A full description of the statistical parameters including central tendency (e.g. means) or other basic estimates (e.g. regression coefficient) AND variation (e.g. standard deviation) or associated estimates of uncertainty (e.g. confidence intervals)
☒	☐	For null hypothesis testing, the test statistic (e.g. *F*, *t*, *r*) with confidence intervals, effect sizes, degrees of freedom and *P* value noted<br>*Give P values as exact values whenever suitable.*
☒	☐	For Bayesian analysis, information on the choice of priors and Markov chain Monte Carlo settings
☒	☐	For hierarchical and complex designs, identification of the appropriate level for tests and full reporting of outcomes
☒	☐	Estimates of effect sizes (e.g. Cohen's *d*, Pearson's *r*), indicating how they were calculated

Our web collection on statistics for biologists contains articles on many of the points above.

Software and code

Policy information about availability of computer code

Data collection	Data was collected from various open-source sources (see Data Availability statement) and preprocessed in Python software (see data analysis). The production cost data is available at https://zenodo.org/record/7701784#.ZBMVXnaZOUk. The BACI harmonized trade dataset and the Mac-Map-HS6 applied tariff dataset are both available from the CEPII website (https://www.cepii.fr/CEPII/en/bdd_modele/bdd_modele.asp). Global gridded population dataset is available from https://earthdata.nasa.gov/data/catalog/sedac-ciesin-sedac-gpwv4-popdens-r11-4.11 whereas global subnational administrative boundaries are available from GADM (https://gadm.org). The global gridded production and yield data is obtained from MAPSPAM (https://mapspam.info/).
Data analysis	All processing and data analysis was performed using Python software (v.3.912). The Dijkstra Algorithm was applied using the iGraph python package (v0.9.8). To produce the maps and figures, the Matplotlib python package was used (v3.5.2) and the geopandas package (v0.10.2). The KMeans clustering analysis was performed with the scikit-learn package (v1.0.2). The code needed to analyse the data and reproduce the figures is provided in Zenodo repository (https://doi.org/10.5281/zenodo.14028713).

For manuscripts utilizing custom algorithms or software that are central to the research but not yet described in published literature, software must be made available to editors and reviewers. We strongly encourage code deposition in a community repository (e.g. GitHub). See the Nature Portfolio guidelines for submitting code & software for further information.

Data

Policy information about availability of data

All manuscripts must include a data availability statement. This statement should provide the following information, where applicable:

- Accession codes, unique identifiers, or web links for publicly available datasets
- A description of any restrictions on data availability
- For clinical datasets or third party data, please ensure that the statement adheres to our policy

The production cost data is available at https://zenodo.org/record/7701784#.ZBMVXnaZOUk. The BACI harmonized trade dataset and the Mac-Map-HS6 applied tariff dataset are both available from the CEPII website (https://www.cepii.fr/CEPII/en/bdd_modele/bdd_modele.asp). Global gridded population dataset is available from https://earthdata.nasa.gov/data/catalog/sedac-ciesin-sedac-gpwv4-popdens-r11-4.11 whereas global subnational administrative boundaries are available from GADM (https://gadm.org). The global gridded production and yield data is obtained from MAPSPAM (https://mapspam.info/). The subnational transport cost dataset and model output to reproduce the analysis is deposited in a Zenodo repository (https://doi.org/10.5281/zenodo.14028713).

Human research participants

Policy information about studies involving human research participants and Sex and Gender in Research.

Reporting on sex and gender	NA
Population characteristics	NA
Recruitment	NA
Ethics oversight	NA

Note that full information on the approval of the study protocol must also be provided in the manuscript.

Field-specific reporting

Please select the one below that is the best fit for your research. If you are not sure, read the appropriate sections before making your selection.

☐ Life sciences ☐ Behavioural & social sciences ☒ Ecological, evolutionary & environmental sciences

For a reference copy of the document with all sections, see nature.com/documents/nr-reporting-summary-flat.pdf

Ecological, evolutionary & environmental sciences study design

All studies must disclose on these points even when the disclosure is negative.

Study description	This study includes extensive data collection effort, bringing together data from publicly available different sources. This is tied together with a new global transport model to estimate the trade costs between subnational regions globally.
Research sample	Sample of countries and their subnational regions.
Sampling strategy	NA
Data collection	NA
Timing and spatial scale	NA
Data exclusions	NA
Reproducibility	NA
Randomization	NA
Blinding	NA

Did the study involve field work? ☐ Yes ☒ No

Reporting for specific materials, systems and methods

We require information from authors about some types of materials, experimental systems and methods used in many studies. Here, indicate whether each material, system or method listed is relevant to your study. If you are not sure if a list item applies to your research, read the appropriate section before selecting a response.

Materials & experimental systems

n/a	Involved in the study
☒	☐ Antibodies
☒	☐ Eukaryotic cell lines
☒	☐ Palaeontology and archaeology
☒	☐ Animals and other organisms
☒	☐ Clinical data
☒	☐ Dual use research of concern

Methods

n/a	Involved in the study
☒	☐ ChIP-seq
☒	☐ Flow cytometry
☒	☐ MRI-based neuroimaging
